# Topological *n*-root Su–Schrieffer–Heeger model in a non-Hermitian photonic ring system

**DOI:** 10.1515/nanoph-2023-0590

**Published:** 2024-01-03

**Authors:** David Viedma, Anselmo M. Marques, Ricardo G. Dias, Verònica Ahufinger

**Affiliations:** Departament de Física, Universitat Autònoma de Barcelona, E-08193 Bellaterra, Spain; Department of Physics & i3N, University of Aveiro, 3810-193 Aveiro, Portugal

**Keywords:** topological photonics, ring resonators, high-root topology, non-Hermitian systems, edge states

## Abstract

Square-root topology is one of the newest additions to the ever expanding field of topological insulators (TIs). It characterizes systems that relate to their parent TI through the squaring of their Hamiltonians. Extensions to 2^*n*^-root topology, where *n* is the number of squaring operations involved in retrieving the parent TI, were quick to follow. Here, we go one step further and develop the framework for designing general *n*-root TIs, with *n* any positive integer, using the Su–Schrieffer–Heeger (SSH) model as the parent TI from which the higher-root versions are constructed. The method relies on using loops of unidirectional couplings as building blocks, such that the resulting model is non-Hermitian and embedded with a generalized chiral symmetry. Edge states are observed at the *n* branches of the complex energy spectrum, appearing within what we designate as a ring gap, shown to be irreducible to the usual point or line gaps. We further detail on how such an *n*-root model can be realistically implemented in photonic ring systems. Near perfect unidirectional effective couplings between the main rings can be generated via mediating link rings with modulated gains and losses. These induce high imaginary gauge fields that strongly suppress couplings in one direction, while enhancing them in the other. We use these photonic lattices to validate and benchmark the analytical predictions. Our results introduce a new class of high-root topological models, as well as a route for their experimental realization.

## 1 Introduction

High-root topology has emerged as a rich new branch within the field of topological insulators (TIs). Square-root TIs $\left(\sqrt{\text{TIs}}\right)$ [1] were first proposed to characterize lattice models whose parent TI, from which its topological features are inherited, manifests itself as one of the diagonal blocks of the squared Hamiltonian [2], [3], [4], [5], [6], [7], [8], [9], [10], [11]. Experimental realization of these models in different platforms followed soon [12], [13], [14], [15], [16], [17], [18], [19], [20]. Subsequently, these systems were further generalized to 2^*n*^-root TIs $\left(\sqrt[{2^{n}}]{\text{TIs}}\right)$ [21], [22], [23], [24], meaning models that connect to their parent TI through a sequence of *n* squaring operations. The first experimental demonstrations of quartic-root topology (*n* = 2) appeared recently in the context of acoustic [25] and photonic [26] lattices. Studies on related topics, such as those of fractionally twisted models [27] or multiplicative topological phases [28], have also started to appear recently.

The question of whether general *n*-root TIs $\left(\sqrt[{n}]{\text{TIs}}\right)$ , with n∈N , can be devised naturally arises. It has already been affirmatively answered for Floquet systems [29], [30], through a method based on subdividing the driving period into *n* subperiods, each with its own associated Hamiltonian. However, for non-driven systems, a natural generalization of $\sqrt[{2^{n}}]{\text{TIs}}$ to $\sqrt[{n}]{\text{TIs}}$ has been lacking so far. Here, we bridge this gap in the literature by considering the SSH model [31] as the parent TI, from which its higher-root versions $\left(\sqrt[{n}]{\text{SSH}}\right)$ are derived following a novel procedure. Specifically, it involves constructing *n*-partite chains from loop modules of unidirectional couplings as the building blocks. Under open boundary conditions (OBC), *n* edge states, all decaying from the same edge [22], are seen to appear in the complex energy spectrum when in the topological phase.

The main challenge regarding the experimental design of the $\sqrt[{n}]{\text{SSH}}$ model relates to the implementation of the unidirectional couplings in the loop modules. Although seemingly exotic, non-Hermitian couplings have been a matter of intense discussion in recent years, with theoretical proposals and experimental implementations appearing in many different platforms, including optical and acoustic ring resonators [7], [32]–[38], optical fibers and waveguides [39]–[41], ultracold atoms [42], [43], electrical circuits [44]–[47], modulated waveguides exploiting synthetic dimensions [48], [49], and many others.

**Figure 1:**
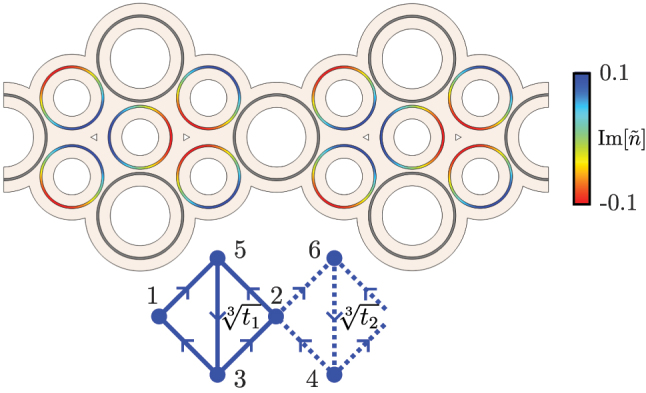
Unit cell geometry of the photonic ring implementation of the $\sqrt[{3}]{\text{SSH}}$ model. The grey rings constitute the main rings of the effective lattice, without gain or loss. The smaller link rings are anti-resonant to the former, and display a sine-like distribution of the imaginary component of the refractive index $\tilde{n}$ , as represented by the color bar on the right. The distance between rings is different in each plaquette so that $\sqrt[{3}]{t_{1}}\neq \sqrt[{3}]{t_{2}}$ . The lower inset depicts the unit cell of the $\sqrt[{3}]{\text{SSH}}$ model, where the arrows indicate the direction of the couplings, and corresponds to an effective description of the counter-clockwise (*m* = −1) circulation of the photonic system above. For the opposite clockwise (*m* = 1) circulation, an equivalent model is obtained, but with all coupling directions flipped.

We focus here on photonic ring systems, and show that they are a well suited candidate for the realization of these models. We consider an array made up of a set of resonant optical ring resonators, which constitute the main rings of the lattice, coupled through smaller anti-resonant link rings, as illustrated in Figure 1. The link rings feature a split gain/loss distribution, in which the upper half of the ring has gain characterized by a parameter *h* while the lower half has an equal amount of loss. To avoid reflection effects, we use a sine-like distribution for the imaginary part of the refractive index. The anti-resonant condition for a ring mode with propagation constant *β* reads: $\beta \left(L_{L}-L_{M}\right)=\left(2m+1\right)\pi$ , where *L*_*M*_ and *L*_*L*_ are the lengths of main and link rings, respectively, and *m* is the circulation. Here, we will restrict ourselves to the clockwise (*m* = 1) and counter-clockwise (*m* = −1) circulations. Through the presence of the link rings, and due to their balanced gain and loss distribution, an effective asymmetric coupling is enabled between the same circulation *m* in the main rings *t*_±_ = *t e* ^±*h*^ [32], which depends exponentially on the gain and loss parameters and is analogous to an imaginary gauge field acting on the system. We represent the forward coupling direction by + and the backward direction by −. Unidirectionality in the couplings is obtained in the limit *h* → ∞, while Hermiticity is restored for *h* = 0. For a finite *h* value, one is in the intermediate situation where the hoppings occur in both directions, but with the predominance of one over the other. For a strong enough gauge field, nearly perfect unidirectionality can be achieved, as we propose below. The coupling *t* is determined by the relative distance between the main rings, which for the roots of the SSH model alternates between two values in different plaquettes to achieve $\sqrt[{3}]{t_{1}}\neq \sqrt[{3}]{t_{2}}$ (this choice of hopping notation [22], [23], [25], adopted from now on, will become clear below, when we relate the model in Figure 1 to its cubed SSH parent system). A key characteristic in this system is that for each pair of rings, only opposite circulations may be coupled between them. That is, we consider that the coupling of the (counter-)clockwise circulation of a main ring with the (counter-)clockwise of a link ring is negligible. In that sense, we can separate the system in clockwise and counter-clockwise components for all main rings. This assumption is valid as long as the coupling region between main and link rings is long compared to the wavelength of light [50], which is fulfilled for the sizes considered in this work and is reflected in the numerical results. Additionally, a real flux of desired value can be established in ring systems by orthogonally displacing a link ring from the line connecting the centers of the corresponding main rings, and thus generating a phase in the coupling between them [51].

We will begin with a brief overview of the $\sqrt[{3}]{\text{SSH}}$ model and its main features. The interested reader is referred to Supplementary Section I for the complete analytical description of the model. Next, we will detail on how such a model can be implemented in a photonic ring system. After a brief discussion on the generalized $\sqrt[{n}]{\text{SSH}}$ model, we will finish with an analysis of the photonic realization of the $\sqrt[{4}]{\text{SSH}}$ model. Numerical simulations on the ring systems are performed using the commercial finite-element simulation software COMSOL Multiphysics. All relevant parameters necessary to reproduce the results are indicated within the main text or Supplementary Material, or can be deduced from them.

## $\sqrt[{3}]{\text{SSH}}$ model

2

The unit cell of the $\sqrt[{3}]{\text{SSH}}$ model is depicted at the bottom of Figure 1. Under periodic boundary conditions (PBC), and in the ordered {|*j*(*k*)⟩} basis, with *j* = 1, 2, *…*, 6, the bulk Hamiltonian of the $\sqrt[{3}]{\text{SSH}}$ model can be written as

(1) $$H_{\sqrt[{3}]{\text{SSH}}}\left(k\right)=\left(\begin{array}{ccc} 0 & h_{1} & 0\\ 0 & 0 & h_{2}\\ h_{3} & 0 & 0 \end{array}\right),$$

(2) $$h_{1}=h_{3}^{†}=-\left(\begin{array}{cc} \sqrt[{3}]{t_{1}} & \sqrt[{3}]{t_{2}}e^{-ik}\\ \sqrt[{3}]{t_{1}} & \sqrt[{3}]{t_{2}} \end{array}\right),$$

(3) $$h_{2}=-\left(\begin{array}{cc} \sqrt[{3}]{t_{1}} & 0\\ 0 & \sqrt[{3}]{t_{2}} \end{array}\right),$$

where the lattice spacing was set to unity and all hopping terms are unidirectional. We further assume *t*_1_, *t*_2_ ≥ 0, without loss of generality. Due to its tripartite nature, composed by the sublattices (1,2), (3,4), and (5,6), and defined by requiring multiples of three hopping processes to produce intra-sublattice couplings [52], this Hamiltonian obeys a generalized chiral symmetry,

(4) $$C_{3}:\;\;\Gamma_{3}H_{\sqrt[{3}]{\text{SSH}}}\left(k\right)\Gamma_{3}^{-1}=\omega_{3}^{-1}H_{\sqrt[{3}]{\text{SSH}}}\left(k\right),$$

(5) $$\Gamma_{3}=\mathrm{diag}\left(\sigma_{0},\omega_{3}\sigma_{0},\omega_{3}^{-1}\sigma_{0}\right),$$

with $\omega_{3}={\text{e}}^{\text{i}\frac{2\pi }{3}}$ and *σ*_0_ the 2 × 2 identity matrix.

After cubing the Hamiltonian in (1) we obtain

(6) $$H_{\sqrt[{3}]{\text{SSH}}}^{3}\left(k\right)=\mathrm{diag}\left(H_{{\text{SSH}}^{\prime }}\left(k\right),H_{2}\left(k\right),H_{3}\left(k\right)\right),$$

where

(7) $$\begin{array}{ll} H_{{\text{SSH}}^{\prime }}\left(k\right) & =h_{1}h_{2}h_{3}\\ & =-\left(\begin{array}{cc} t_{1}+t_{2} & t_{1}+t_{2}e^{-ik}\\ t_{1}+t_{2}e^{ik} & t_{1}+t_{2} \end{array}\right)\\ & =-\left(t_{1}+t_{2}\right)\sigma_{0}+H_{\text{SSH}}\left(k\right), \end{array}$$

is isospectral to the other diagonal terms in (6), namely *H*_2_(*k*) = *h*_2_ *h*_3_ *h*_1_ and *H*_3_(*k*) = *h*_3_ *h*_1_ *h*_2_ [52]. Their eigenvalues are given by

(8) $$E_{\pm }\left(k\right)=-t_{1}-t_{2}\pm \sqrt{t_{1}^{2}+t_{2}^{2}+2t_{1}t_{2}\cos k}.$$

**Figure 2:**
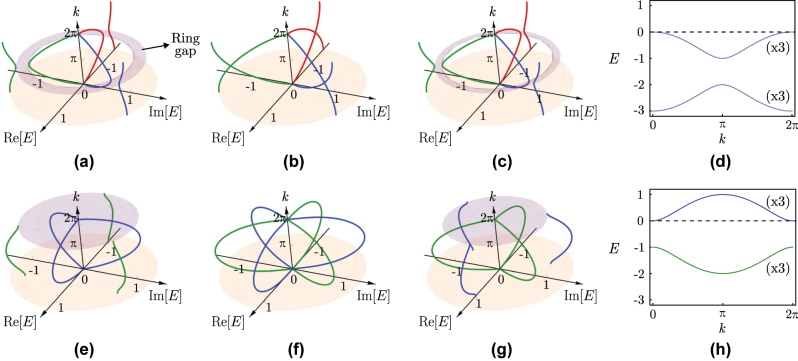
Complex energy spectrum, in units of $\sqrt[{3}]{t_{2}}=1$ , as a function of the momentum for the $\sqrt[{3}]{\text{SSH}}$ model in (1) with (a) $\sqrt[{3}]{t_{1}}=\sqrt[{3}]{0.5}$ , (b) $\sqrt[{3}]{t_{1}}=1$ , and (c) $\sqrt[{3}]{t_{1}}=\sqrt[{3}]{1.5}$ . Different energy branches are indicated with different colors. (d) Cubed energy spectrum of the model in (a), which is purely real and with each band three-fold degenerate. (e)–(h) Same as the corresponding cases above, but for $\sqrt[{3}]{t_{1}}\to {\text{e}}^{\text{i}\frac{\pi }{3}}\sqrt[{3}]{t_{1}}$ in (1), leading to the ${\sqrt[{3}]{\text{SSH}}}_{\frac{\pi }{3}}$ model. Different colors in (e)–(g) now distinguish the groups of three bands that become degenerate upon cubing the spectrum. The ring gaps are depicted in light purple and appear at *k* = *π* in (a) and (c), and at *k* = 2*π* in (e) and (g), where the inner circumference of the ring reduces to a point at *E* = 0.

The three-fold degenerate spectrum of Figure 2(d) is a reflection of the isospectrality of the three diagonal blocks.

The complex energy spectrum of the $\sqrt[{3}]{\text{SSH}}$ model, with the Hamiltonian in (1), is composed of three two-band branches that can be derived directly from (8) as $\left\{E_{\pm }^{\frac{1}{3}}\left(k\right),\omega_{3}E_{\pm }^{\frac{1}{3}}\left(k\right),\omega_{3}^{-1}E_{\pm }^{\frac{1}{3}}\left(k\right)\right\}$ . In Figure 2(a)–(c), we represent this bulk energy spectrum for different values of $\sqrt[{3}]{t_{1}}$ , after setting $\sqrt[{3}]{t_{2}}=1$ . The three branch structure, indicated by the different colors, is clearly visible. From one branch, the other two can be obtained from successive $\frac{2\pi }{3}$ rotations in the complex energy plane, as a consequence of the $C_{3}$ -symmetry in (4). Additionally, the low energy bands of the three branches become degenerate at *E* = 0 for *k* = 0. As we detail in Supplementary Section I, this actually corresponds to an exceptional point of the spectrum, with only two associated eigenstates. The downshifted three-fold degenerate SSH real spectrum of Figure 2(d) was obtained by cubing the complex spectrum of the $\sqrt[{3}]{\text{SSH}}$ model in Figure 2(a).

Remarkably, the spectral gap for the $\sqrt[{3}]{\text{SSH}}$ model does not fall in the conventional categories of non-Hermitian systems, namely those of point or line gaps [53], [54], which are present if the Hamiltonian can be continuously flattened into a unitary matrix without closing the respective gap. Here, and since the starting $\left(\sqrt[{3}]{\text{SSH}}\right)$ model is directly related to the parent (SSH) model by a cubing procedure, the energy gap of the latter [see Figure 2(d)], present for *t*_1_ ≠ *t*_2_, naturally reverts back to all three branches of the complex spectrum [see Figure 2(a)]. This generates what we label as a *ring gap* in the energy spectrum, not reducible to a point or a line gap. In the sequence of Figure 2(a)–(c), we can see the ring gap closing and reopening across the critical point $\sqrt[{3}]{t_{1}}=\sqrt[{3}]{t_{2}}$ . A continuous ring gap is obtained in the *n* → ∞ limit of the $\sqrt[{n}]{\text{SSH}}$ model studied below, corresponding to an infinite number of branches forming a continuum in the energy spectrum (see the energy spectrum of the *n* = 20 case in Supplementary Section II).

**Figure 3:**
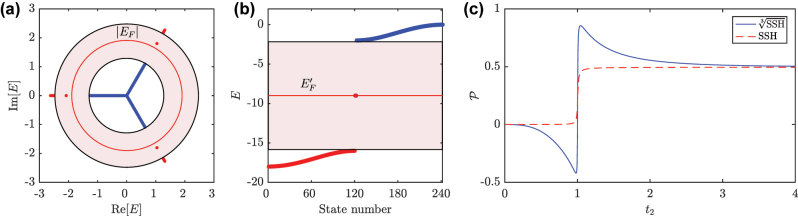
Computing the polarization in the root and parent systems. (a) Complex energy spectrum of the open $\sqrt[{3}]{\text{SSH}}$ chain with *N* = 121 unit cells for $\sqrt[{3}]{t_{1}}=1$ and $\sqrt[{3}]{t_{2}}=2$ , with the ring gap highlighted in pink. The ring Fermi level of radius |*E*_*F*_| is marked with a solid red line. (b) Energy spectrum of an open SSH chain of the same size *N*, with lattice parameters extracted from the corresponding diagonal block of the cubed Hamiltonian of the model in (a), with the Fermi level $E_{F}^{\prime }$ placed at half-filling, where small edge perturbations were included to place the right (left) in-gap edge state below (above) the Fermi level. (c) Polarization in the $\sqrt[{3}]{\text{SSH}}$ (blue solid line) and the SSH (red dashed line) models, as a function of *t*_2_ for *t*_1_ = 1, computed by filling the states marked in red in (a) and (b), respectively.

We can take advantage of the existence of a ring energy gap in the system to define a new type of polarization for our complex spectra. The polarization is computed by filling all states below a certain Fermi level, which is not well defined for complex spectra. In our case, we introduce a *ring Fermi level* at a certain radius |*E*_*F*_| within the ring gap, such that all states within it are considered occupied and unoccupied otherwise. However, and for the purpose of comparing with the polarization of the parent SSH model, we will rather occupy the states outside the Fermi level, as depicted in Figure 3(a) for an open chain in the topological phase. This is justified by considering that, when cubing the root model, the outer bands become the degenerate lower energy band of the SSH model, as we highlight in Figure 3(b). This can also be understood by comparing Figure 2(a)–(d). In this vein, we define the polarization as:

(9) $$P=\frac{e}{N}\overset{\frac{N-1}{2}}{\underset{j=-\frac{N-1}{2}}{\sum }}\overset{6}{\underset{\alpha =1}{\sum }}\overset{6N}{\underset{l=3N+1}{\sum }}j{\left(\Psi_{j,\alpha ,l}^{L}\right)}^{*}\Psi_{j,\alpha ,l}^{R},$$

where *e* is the electron charge, *j* the unit cell position, *α* the site number within the unit cell, *l* the eigenstate index, and $\Psi_{j,\alpha ,l}^{L\left(R\right)}$ the amplitude of the left (right) eigenstate *l* of the root model at the corresponding position. The process leading to expression (9) is detailed in Supplementary Section III. We plot the polarization for an open $\sqrt[{3}]{\text{SSH}}$ chain of *N* = 121 unit cells at half-filling in Figure 3(c) (blue solid line), as a function of *t*_2_ for *t*_1_ = 1. We see that the polarization, in units of *e*, is quantized only at the atomic limits, $P\left(t_{2}=0\right)=0$ and $P\left(t_{2}\to \infty \right)=1/2$ , while being non-quantized away from these limits, with a sharp transition at the critical gap closing point *t*_2_ = 1. This behavior is in agreement with that of the $\sqrt{\text{SSH}}$ model [55] where, analogously, the polarization values are non-quantized across the parameters range, whereas the difference between the atomic limits remains quantized to 1/2, as observed here also. It is this quantized polarization difference between atomic limits, located at opposite sides of the transition point *t*_2_ = 1, that serves as a topological invariant of our root systems. For comparison, we plot in the same Figure 3(c) the usual polarization at half-filling for the parent SSH chain of the same size *N* (red dashed line). There, the usual quantized plateaus at P=0 (P=1/2) for the (non-)trivial region and the transition from one to the other at the gap closing point are observed, once more demonstrating that the parent SSH model is indeed the source of the topological features of the root models. More details on the physical interpretation of the polarization at both atomic limits (*t*_1_ = 0 and *t*_2_ = 0) are given in Supplementary Section III.

**Figure 4:**
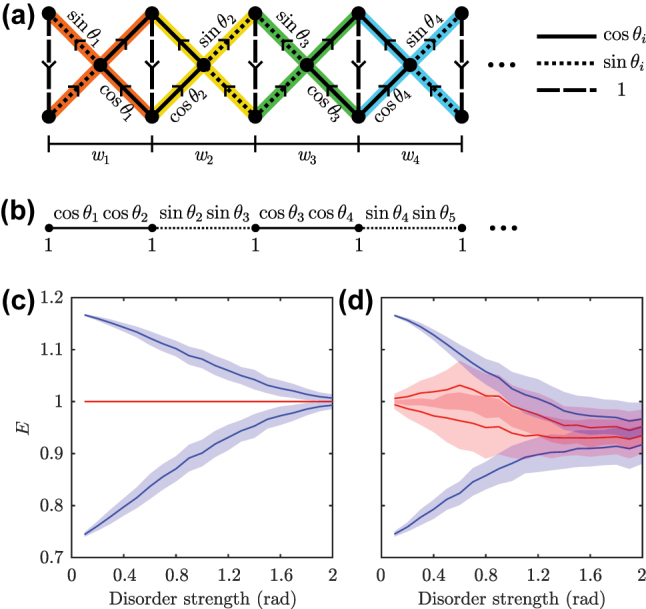
Correlated and uncorrelated disorder. (a) Sketch of a $\sqrt[{3}]{\text{SSH}}$ model with correlated disorder *θ*_*i*_ = *θ*_*c*_ + *w*_*i*_ sampled in quartets of hopping terms, as indicated by the lower segments and the different shaded colors in the hoppings, from a uniform distribution $w_{i}\in \left[-W/2,W/2\right]$ , and with the vertical couplings being disorder free. (b) Sketch of the Hermitian SSH system obtained for the decoupled spinal sublattice when cubing the Hamiltonian corresponding to (a). Due to the correlated disorder in (a), this system displays only off-diagonal disorder. (c), (d) Mean value (solid lines) and standard deviation (shaded region) for the edge states (red) and the closest bulk states (blue) in the real branch of the disordered root system for increasing strengths of (c) correlated and (d) uncorrelated disorder, taken over 200 different realizations.

We now focus on the topological edge states that can be found within the ring gap. As described in previous works [22], [23], the root model inherits the topological protection from the parent system, which in our case is the Hermitian SSH model. In that sense, the edge states of the $\sqrt[{3}]{\text{SSH}}$ model are protected against any disorder that preserves *the chiral symmetry of the parent SSH model*, which is the protecting symmetry. As is well known, the SSH model is protected against chiral disorder such as off-diagonal disorder. As such, we expect our edge states to be protected against the types of disorder in the $\sqrt[{n}]{\text{SSH}}$ systems that, when raised to the *n*th-power, translate as chiral disorder for the parent SSH block. This constitutes a smaller subset of allowed disorders when compared to those of the parent SSH model, which has been characterized as a dilution of the topological protection [22]. An example of such disorder is provided in Figure 4(a), where we have added two extra sites to the left of the chain to impose inversion symmetry. In there, we consider disorder around $\sqrt[{3}]{t_{1}}=\cos\theta_{c}$ and $\sqrt[{3}]{t_{1}}=\sin\theta_{c}$ , where *θ*_*c*_ is defined so that $\sqrt[{3}]{t_{2}}/\sqrt[{3}]{t_{1}}=2$ and two edge states per branch appear, located at opposite edges. As indicated in the figure, the disorder is sampled from a uniform distribution in quartets of hopping terms, and is thus correlated. When cubing this system, the SSH block takes the form sketched in Figure 4(b), where the onsite energies are sin^2^ *θ*_*i*_ + cos^2^ *θ*_*i*_ = 1 and the disorder is off-diagonal. The response of the edge and closest bulk states to this disorder is presented in Figure 4(c), where it is clear that the edge states remain unaltered until the disorder is strong enough to close the gap. As a direct comparison, we provide the same plot for uncorrelated disorder where each *θ*_*i*_ is sampled independently in Figure 4(d), which corresponds to both onsite and off-diagonal disorder for the diagonal SSH block when cubed. For this case, it can be seen that the robustness of the edge states is lost. This proves that the topological protection in our model is related to the preservation of the chiral symmetry of the parent SSH model. To further visualize these results, we provide animations in the Supplementary Material showing different realizations of correlated and uncorrelated disorder. There, one can readily see that the whole spectrum is affected by the disorder, and only for the case of correlated disorder do the edge states remain unaltered.

Another interesting effect occurs when a *π* magnetic flux is uniformly distributed in the loops of the rhombus with one type of hopping term in each unit cell. For example, let us consider the Peierls substitution $\sqrt[{3}]{t_{1}}\to {\text{e}}^{\text{i}\frac{\pi }{3}}\sqrt[{3}]{t_{1}}$ at the unit cell shown at the bottom of Figure 1. We label the resulting system as the ${\sqrt[{3}]{\text{SSH}}}_{\frac{\pi }{3}}$ model. As explained in more detail in Supplementary Section I, and illustrated in Figure 2(e)–(g), when this change is included in the Hamiltonian in (1), it induces a $\frac{\pi }{3}$ relative rotation in the complex plane between the three outer bands and the three inner bands, and we lose the one-to-one correspondence between an outer and an inner band that previously defined each branch. Instead, now we distinguish between outer and inner branches, which have a relative $\frac{\pi }{3}$ phase difference.

Notice that the cubed spectrum of Figure 2(h) is shifted up, in relation to the one in Figure 2(d), such that one of the three-fold degenerate bands is pushed to the positive half of the spectrum, and also that there is a relative *π* sliding of the bands between the two cases. The energy gap is open now at *k* = 0 in Figure 2(h). Therefore, the spectral gap of the corresponding ${\sqrt[{3}]{\text{SSH}}}_{\frac{\pi }{3}}$ child model in Figure 2(e) is also defined as a ring gap at the *k* = 0 point, which is the gap closing point in Figure 2(f), with the inner circumference of the ring reduced to a single degenerate point at zero energy. However, the ring gap at *k* = 0 gets obscured if one employs the usual projection of the whole spectrum onto the complex energy plane [e.g., the ring gaps of Figure 2(e)–(g) are clearly not visible upon projecting the spectrum onto the energy plane]. This shows how the three-dimensional representation of the spectrum, as in Figure 2(e)–(g), is required for the manifestation of the ring gap in the ${\sqrt[{3}]{\text{SSH}}}_{\frac{\pi }{3}}$ model.

## Photonic ring realization of the $\sqrt[{3}]{\text{SSH}}$ model

3

**Figure 5:**
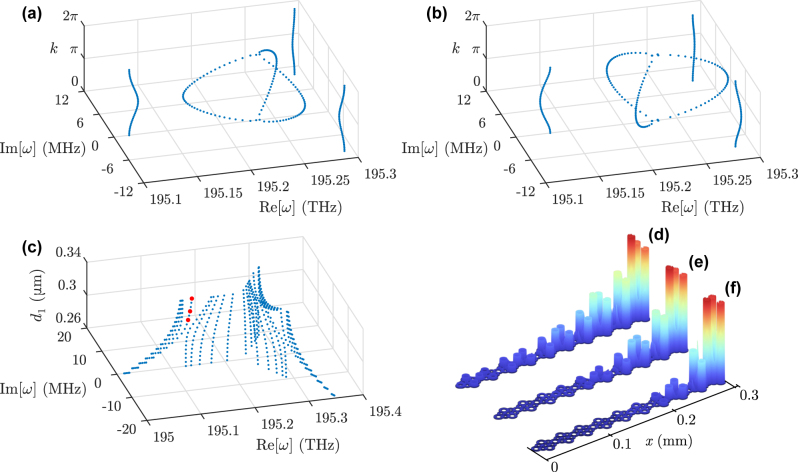
Eigenfrequencies of the photonic (a) $\sqrt[{3}]{\text{SSH}}$ model and (b) ${\sqrt[{3}]{\text{SSH}}}_{\frac{\pi }{3}}$ model with PBC at steps of Δ*k* = 0.05*π*. The three-fold splitting along the complex plane can be readily observed. (c) Eigenspectrum of the photonic $\sqrt[{3}]{\text{SSH}}$ chain with OBC and *N* = 5 unit cells, with *d*_2_ = 0.3 μm and *d*_1_ spanning both the topologically trivial and nontrivial phases, i.e., *d* ∈ [0.26 μm, 0.34 μm]. Three sets of edge modes appear along the ring gap. (d)–(f) Electric field norms for the edge modes marked in red in (c), for (d) *d*_1_ = 0.315 μm, (e) *d*_1_ = 0.325 μm, and (f) *d*_1_ = 0.34 μm, respectively.

We consider rings of planar waveguides with a radius of 4.5 μm and a width of 250 nm. For simplicity, the cores (with refractive index ${\tilde{n}}_{\text{core}}=3$ ) are surrounded by air, leading to an overall high contrast, and the considered resonant frequency is 195.225 THz. The asymmetric effective coupling is established through smaller anti-resonant link rings of radius 3.24 μm and core refractive index ${\tilde{n}}_{\text{link}}=3+0.1i\sin\varphi$ , where *φ* is the angle of the polar coordinates with origin at the center of the link ring. For these parameter values, the loss factor in *t*_±_ = *t e* ^±*h*^ is computed to be *h* = 2.07, implying a coupling asymmetry ratio of *α* ≡ *t*_−_/*t*_+_ = 0.016, very close to perfect unidirectionality. Proof that this ring setup generates such a coupling is provided in Supplementary Section IV. We display the unit cell of the system in Figure 1, where we have considered the counter-clockwise circulation (*m* = −1) for the coupling distribution shown in the inset. To achieve the staggered distribution of couplings present in the $\sqrt[{3}]{\text{SSH}}$ model, this structure is replicated with alternating relative ring distances *d*_1_ = 0.33 μm and *d*_2_ = 0.3 μm in each plaquette, which corresponds to a coupling ratio of $\sqrt[{3}]{t_{1}}/\sqrt[{3}]{t_{2}}≃0.6$ . These distances are drawn between the outer radii of the rings. The method to extract the couplings and the asymmetry parameter *h* from the spectrum of the ring resonators is described in Supplementary Sections V and VI, respectively. We display the bulk spectrum of eigenfrequencies for the periodic ring system in Figure 5(a). The spectrum agrees very well with the theoretical results shown in Figure 2(a). As explained above, the three-fold splitting in the complex energy plane is a consequence of the tripartite nature of the system, as it obeys the generalized chiral symmetry in (4). As before, the band gap for this system can be generalized to the complex frequency spectrum context by defining a ring gap for all |*ω*| in a certain interval, a concept that can be directly applied to higher-root systems as well, as we will show below. On a separate note, although the spectra for both circulations in the main rings are obtained in the simulations, we only observe a doubly-degenerate joint spectrum. Reversing the circulation in the rings corresponds to a change of all coupling directions, but the cubed system in that case is still the SSH model. This necessarily implies that both circulations yield the same spectrum, as explained in Ref. [52]. The symmetries of the full $\sqrt[{3}]{\text{SSH}}$ system, which incorporates both *m* = ±1 circulations, are further detailed in Supplementary Section IB.

A real flux can be added to the system by displacing the link rings orthogonally to the coupling line [51], which modifies the optical path in the upper and lower arms and induces a phase in the coupling, as shown in Supplementary Section VI. We are particularly interested in realizing the ${\sqrt[{3}]{\text{SSH}}}_{\frac{\pi }{3}}$ model by considering a *π* flux around the loops involving one type of hopping terms, namely by considering the following Peierls substitution, $\sqrt[{3}]{t_{1}}\to {\text{e}}^{\text{i}\frac{\pi }{3}}\sqrt[{3}]{t_{1}}$ . As detailed in the previous section, this implies a sign change for one of the bands of the parent SSH model. Relative to the $\sqrt[{3}]{\text{SSH}}$ model of Figure 5(a), we can see in Figure 5(b) that the flux causes a *π*-sliding of the outer bands and a *π*/3 rotation of the inner ones, again in agreement with the theoretical results of Figure 2(e).

As one would expect from a root TI, the existence of edge states in the $\sqrt[{3}]{\text{SSH}}$ model under OBC is inherited from the parent system. One of the remarkable features of the cubic-root system is that, since it possesses three times as many bands as the parent one, it will host three times as many in-gap states at one of the edges, namely the right one. The absence of topological states at the left edge can be understood as follows. Upon cubing the lattice, the resulting SSH chain at the first sublattice will have an onsite energy offset at the leftmost site, due to its lower coordination number at the cubic-root level (two missing connections at its left). This onsite energy shift converts the left edge state into a bulk state (the converse reasoning applies to the other two pseudo-Hermitian residual chains of the cubed model, that is, it is their respective perturbations at the right edge sites that drive the formation of an in-gap state there). This mechanism of single-edge locking of the topological modes is typical of high-root TIs, as demonstrated, e.g., for the diamond chain (a square-root model) in [22]. For a lattice of *N* = 5 unit cells, keeping the relative distances in one sublattice fixed at *d*_2_ = 0.3 μm and sweeping *d*_1_ across the topological transition point yields the spectrum showcased in Figure 5(c). The edge states are exponentially localized around one of the ends of the lattice, with the localization length growing as *d*_1_ gets closer to *d*_2_ and the states evolving into bulk states after crossing the critical point *d*_1_ = *d*_2_, that is, after crossing to the topologically trivial regime. Three examples for different *d*_1_ are shown in Figure 5(d)–(f), corresponding to the eigenfrequencies marked in red in Figure 5(c). The edge states from the other two branches are also localized around the same end of the chain, albeit with different phases in the main rings.

## $\sqrt[{n}]{\text{SSH}}$ model

4

**Figure 6:**
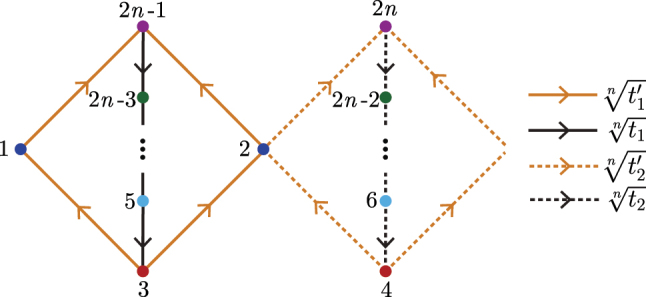
Unit cell of the $\sqrt[{n}]{\text{SSH}}$ model, composed of 2*n* sites and *n* sublattices, indicated by different colors, of two sites each. The arrows indicate the hopping direction, with the hopping terms assumed unidirectional. Without loss of generality, the hopping terms to or from the spinal dark blue sublattice sites can be different from the rung ones, as will be the case with the photonic ring systems studied in Section 4. As in the cubic-root case of Figure 2(e)–(g), a $\frac{\pi }{n}$ phase shift between the two branches of *n* bands in the complex energy spectrum can be obtained with the Peierls substitution $\sqrt[{n}]{t_{i}^{\left(\prime \right)}}\to \sqrt[{n}]{t_{i}^{\left(\prime \right)}}{\text{e}}^{\text{i}\frac{\pi }{n}}$ , with *i* = 1 ∨ 2.

As detailed in Supplementary Section II, our method can be generalized to produce higher-root versions of the SSH parent model, in what we designate as the $\sqrt[{n}]{\text{SSH}}$ model, with integer *n* > 3. The unit cell of this system is depicted in Figure 6. The bulk Hamiltonian of this system, $H_{\sqrt[{n}]{\text{SSH}}}\left(k\right)$ , exhibits *n* two-band branches in its complex energy spectrum after diagonalization, with each branch separated from the next by a $\frac{2\pi }{n}$ angle in the energy plane due to the generalized chiral symmetry,

(10) $$C_{n}:\;\;\Gamma_{n}H_{\sqrt[{n}]{\text{SSH}}}\left(k\right)\Gamma_{n}^{-1}=\omega_{n}^{-1}H_{\sqrt[{n}]{\text{SSH}}}\left(k\right),$$

with the Γ_*n*_ operator given in Supplementary Section II and $\omega_{n}={\text{e}}^{\text{i}\frac{2\pi }{n}}$ . After computing $H_{\sqrt[{n}]{\text{SSH}}}^{n}\left(k\right)$ , one obtains the Hamiltonian of the SSH model as one of its *n* isospectral diagonal blocks. Under OBC and for an integer number of unit cells, the $\sqrt[{n}]{\text{SSH}}$ model hosts *n* edge states for the topologically non-trivial phase *t*_1_ < *t*_2_, appearing at the energy gap between the two bands of each branch, which globally define the ring gap of the system. Finally, in the same way that as one can change from the $\sqrt[{3}]{\text{SSH}}$ to the ${\sqrt[{3}]{\text{SSH}}}_{\frac{\pi }{3}}$ model by introducing a $\frac{\pi }{3}$ Peierls phase at one of the hopping types (shown in Section 2), the ${\sqrt[{n}]{\text{SSH}}}_{\frac{\pi }{n}}$ model can be realized by applying to the hoppings in Figure 6 the transformation $\sqrt[{n}]{t_{j}^{\left(\prime \right)}}\to \sqrt[{n}]{t_{j}^{\left(\prime \right)}}{\text{e}}^{\text{i}\frac{\pi }{n}}$ , with *j* = 1 ∨ 2. Analogously, the complex energy spectrum of the ${\sqrt[{n}]{\text{SSH}}}_{\frac{\pi }{n}}$ model will display a global $\frac{\pi }{n}$ relative shift between the inner and outer sets of energy bands, as we show below for *n* = 4 and in Supplementary Section IIA for *n* = 5.

Implementing higher-order roots in photonic ring setups can be achieved without a significant increase in complexity by adding additional main rings to the vertical coupling link. Nonetheless, the geometrical constraints forces one to use two different elongated link rings, which are otherwise equivalent to the circular link rings in the previous section. Namely, they are anti-resonant to the main rings and have a distribution of gain and loss, with maximum values that are balanced so that the non-reciprocity ratios are approximately equal in all couplings.

**Figure 7:**
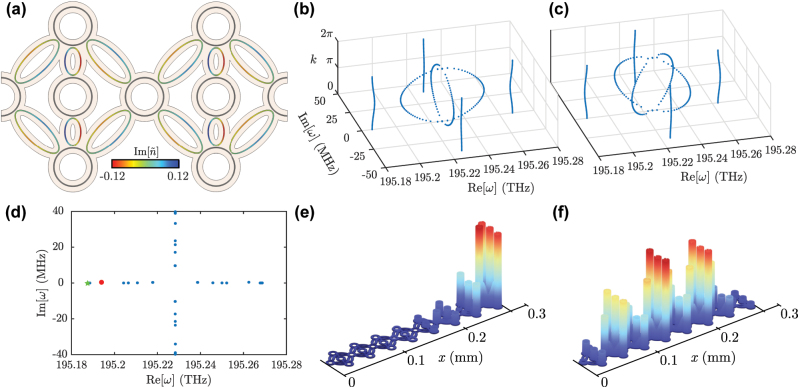
Implementation of the $\sqrt[{4}]{\text{SSH}}$ model. (a) Unit cell for the $\sqrt[{4}]{\text{SSH}}$ model. Shorter (longer) link resonators display stronger (weaker) gain and loss modulations. Eigenfrequencies of the photonic (b) $\sqrt[{4}]{\text{SSH}}$ model and (c) ${\sqrt[{4}]{\text{SSH}}}_{\frac{\pi }{4}}$ model with PBC at steps of Δ*k* = 0.05*π*. (d) Eigenfrequencies of the photonic $\sqrt[{4}]{\text{SSH}}$ chain with OBC and *N* = 4 unit cells, for *d*_1_ = 0.33 μm and *d*_2_ = 0.3 μm, where the four-fold splitting of the bands can be readily observed. (e), (f) Electric field norms for the edge and bulk modes of the system indicated by the red point and green star, respectively, in (d).

In the case of the $\sqrt[{4}]{\text{SSH}}$ model, the unit cell has the shape displayed in Figure 7(a), where the long link rings are of elliptical shape with semiaxis lengths *R*_*a*1_ = 6.85 μm and *R*_*b*1_ = 2.5 μm and maximum loss value of $\text{Im}\left({\tilde{n}}_{\text{link}}\right)=0.072$ . The short ring has semiaxis lengths *R*_*a*2_ = 3.2 μm and *R*_*a*2_ = 1.81 μm and maximum loss value of $\text{Im}\left({\tilde{n}}_{\text{link}}\right)=0.12$ . We use *d*_1_ = 0.33 μm and *d*_2_ = 0.3 μm as alternating distances for both kinds of link rings in each plaquette. This leads to the following coupling values, using the notation indicated in Figure 6 and in units of $\sqrt[{4}]{t_{2}}$ : $\sqrt[{4}]{t_{1}}=0.615$ , $\sqrt[{4}]{t_{1}^{\prime }}=0.566$ and $\sqrt[{4}]{t_{2}^{\prime }}=0.918$ . All these couplings have a non-reciprocity ratio of around *α* = 0.032. With these parameters, we simulate the system both under PBC and OBC. In Figure 7(b), we show that the bulk spectrum of the photonic implementation of the $\sqrt[{4}]{\text{SSH}}$ model correctly captures the four-fold splitting of the bands along the complex plane, as well as the ring gap between the inner and outer bands. A similar agreement with the theoretical result is seen in Figure 7(c), where the bulk spectrum of the photonic ${\sqrt[{4}]{\text{SSH}}}_{\frac{\pi }{4}}$ is plotted. Finally, the spectrum for OBC of the photonic $\sqrt[{4}]{\text{SSH}}$ model with *N* = 4 unit cells is shown in Figure 7(d), where four edge modes are present, as expected. The highlighted edge mode in red is showcased in Figure 7(e), together with a bulk mode in Figure 7(f) for comparison.

## 5 Conclusions

We have demonstrated a method to obtain general *n*-root systems of the SSH model, which requires the usage of unidirectional couplings to be implemented. This poses a challenge, as non-Hermitian systems have proven to be elusive to experimental efforts until recently, where major advances have been achieved [7], ]. Among different possible platforms, we focused on a system of photonic ring resonators, showing it to be a very viable candidate for the implementation of *n*-root TIs, since quasi-unidirectional couplings can be realized by means of auxiliary link rings with a non-uniform imaginary component of the refractive index. Additionally, the high versatility of this platform makes it ideal for designing *n*-root systems, as it also allows, e.g., for a very precise control over the effective magnetic flux piercing the loops of these systems by simply adjusting the position of the link rings.

Implementation of systems similar to the one in this work has been accomplished with waveguide technology [51], where the positioning of the link rings is precise enough to allow introducing real phases in the couplings between main rings. The key challenge in our case is the correct engineering of the link rings. Non-Hermitian couplings in ring systems have already been achieved in lossy acoustic setups [34], [35], [36]. If no gain is considered in our system, or if gain and loss are not perfectly balanced, the effective Hamiltonian picks up imaginary diagonal elements that distort the bands. However, the main features of the model remain unaltered. We showcase this in Supplementary Section VII.

More recently, the split gain and loss has been implemented using optically pumped waveguides, where the lasing of different modes has been exploited [37]. The effective coupling generated in that case is analogous to the one employed here, and could allow to build the root systems in an experiment. Note that the gain/loss function need not be sine-like to achieve the results in this work, although sharp transitions from gain to loss within the same ring may cause reflection effects leading to small cross-circulation couplings. This effect can cause small band splitting, but it does not distort the properties of the whole system. Note that instead one might separate the gain and loss regions into different link rings instead of within a single ring [36], or consider elongated waveguides as couplers over which the available gain can be maximized [37], [38].

On the theoretical side, the method for the construction of *n*-root TIs, based on coupling loop modules of unidirectional couplings, is completely general and therefore not limited to the SSH model. As such, our work paves the way for further studies generalizing the applicability of the method to other emblematic topological and flat-band systems, and is expected to significantly broaden the scope of high-root topology from the 2^*n*^-root models [21], [22], [23], [24] studied thus far.

## Supplementary Material

Supplementary Material Details

Supplementary Material Details

Supplementary Material Details
