# Supplementary material for: Topological n-root Su–Schrieffer–Heeger model in a non-Hermitian photonic ring system
Source: Nanophotonics. 2024 Jan 3;13(1):51–61. doi: 10.1515/nanoph-2023-0590 (PMC11501127; doi:10.1515/nanoph-2023-0590)
Supplement: Supplementary file 1 — Supplementary Material Details [file j_nanoph-2023-0590_suppl_001.pdf]

Supplementary Information for
Topological n-root Su-Schrieffer-Heeger model in a non-Hermitian photonic ring system

David Viedma,[1,*] Anselmo M. Marques,[2] Ricardo G. Dias,[2] and Verònica Ahufinger[1]

[1]*Departament de Física, Universitat Autònoma de Barcelona, E-08193 Bellaterra, Spain*
[2]*Department of Physics & i3N, University of Aveiro, 3810-193 Aveiro, Portugal*

I. CUBIC-ROOT SSH

In the following, we recover and further develop the summarized analytical results on the cubic-root Su-Schrieffer-Heeger ($\sqrt[3]{\text{SSH}}$) model in Section 2 of the main text. In the ordered $\{|j(k)\rangle\}$ basis, with $j = 1, 2, \dots, 6$, the bulk Hamiltonian of the $\sqrt[3]{\text{SSH}}$ model, with the unit cell given in the lower inset of Fig. 1, can be written as

$$H_{\sqrt[3]{\text{SSH}}}(k) = \begin{pmatrix} 0 & h_1 & 0 \\ 0 & 0 & h_2 \\ h_3 & 0 & 0 \end{pmatrix}, \tag{S1}$$

$$h_1 = h_3^\dagger = -\begin{pmatrix} \sqrt[3]{t_1} & \sqrt[3]{t_2}e^{-ik} \\ \sqrt[3]{t_1} & \sqrt[3]{t_2} \end{pmatrix}, \tag{S2}$$

$$h_2 = -\begin{pmatrix} \sqrt[3]{t_1} & 0 \\ 0 & \sqrt[3]{t_2} \end{pmatrix}, \tag{S3}$$

where the lattice spacing was set to unity. Due to its tripartite nature, defined by the sublattices $(1, 2)$, $(3, 4)$ and $(5, 6)$ [1], this Hamiltonian obeys a generalized chiral symmetry,

$$\mathscr{C}_3 : \ \Gamma_3 H_{\sqrt[3]{\text{SSH}}}(k)\Gamma_3^{-1} = \omega_3^{-1} H_{\sqrt[3]{\text{SSH}}}(k), \tag{S4}$$

$$\Gamma_3 = \text{diag}(\sigma_0, \omega_3\sigma_0, \omega_3^{-1}\sigma_0), \tag{S5}$$

with $\omega_3 = e^{i\frac{2\pi}{3}}$ and σ_0 the 2×2 identity matrix.

After cubing the Hamiltonian in (S1) we obtain

$$H^3_{\sqrt[3]{\text{SSH}}}(k) = \begin{pmatrix} H_{\text{SSH}'}(k) & 0 & 0 \\ 0 & H_2(k) & 0 \\ 0 & 0 & H_3(k) \end{pmatrix}, \tag{S6}$$

where

$$\begin{aligned} H_{\text{SSH}'}(k) &= h_1 h_2 h_3 \\ &= -\begin{pmatrix} t_1 + t_2 & t_1 + t_2 e^{-ik} \\ t_1 + t_2 e^{ik} & t_1 + t_2 \end{pmatrix} \\ &= -(t_1 + t_2)\sigma_0 + H_{\text{SSH}}(k), \end{aligned} \tag{S7}$$

whose eigenstates and eigenvalues [2, 3] can be readily determined as

$$E_\pm(k) = -t_1 - t_2 \pm \sqrt{t_1^2 + t_2^2 + 2t_1 t_2 \cos k}, \tag{S8}$$

$$\left|u^\pm_{\text{SSH}}(k)\right\rangle = \frac{1}{\sqrt{2}} \begin{pmatrix} 1 \\ \mp e^{i\phi(k)} \end{pmatrix}, \tag{S9}$$

$$\cot\phi(k) = \frac{t_1}{t_2 \sin k} + \cot k, \tag{S10}$$

* david.viedma@uab.cat

and

$$H_2(k) = h_2 h_3 h_1$$
$$= -\begin{pmatrix} 2t_1 & t_1^{\frac{2}{3}} t_2^{\frac{1}{3}}(1+e^{-ik}) \\ t_1^{\frac{1}{3}} t_2^{\frac{2}{3}}(1+e^{ik}) & 2t_2 \end{pmatrix}, \quad \text{(S11)}$$

and finally

$$H_3(k) = h_3 h_1 h_2 = H_2^\dagger(k). \quad \text{(S12)}$$

It can be proven [1] that all diagonal blocks in (S6) share the same real energy spectrum given in (S8), such that $H_2(k)$ and $H_3(k)$ are pseudo-Hermitian matrices [4] defined as

$$\eta H_2(k)\eta^{-1} = H_2^\dagger(k) = H_3(k), \quad \text{(S13)}$$
$$\eta = \text{diag}(t_1^{-\frac{1}{3}}, t_2^{-\frac{1}{3}}), \quad \text{(S14)}$$

where (S12) was used and $t_1, t_2 > 0$ is assumed. The *finite energy* eigenvectors of the $H_2(k)$ and $H_3(k)$ blocks can be expressed in terms of the $\left|u_{\text{SSH}}^{\pm}(k)\right\rangle$ in (S9) [1],

$$\left|u_{2,R}^{\pm}(k)\right\rangle = \frac{1}{\mathcal{N}_{2,R}^{\pm}(k)} h_2 h_3 \left|u_{\text{SSH}}^{\pm}(k)\right\rangle, \quad \text{(S15)}$$
$$\left|u_{3,R}^{\pm}(k)\right\rangle = \frac{1}{\mathcal{N}_{3,R}^{\pm}(k)} h_3 \left|u_{\text{SSH}}^{\pm}(k)\right\rangle, \quad \text{(S16)}$$

where $\mathcal{N}_{\mu,R}^{\pm(k)}$, with $\mu = 2, 3$, is a normalization constant, and R stands for right eigenvector. The non-Hermiticity of $H_\mu(k)$ implies that its left (L) and right (R) eigenstates, defined as

$$H_\mu(k)\left|u_{\mu,R}^{\pm}(k)\right\rangle = E_\pm(k)\left|u_{\mu,R}^{\pm}(k)\right\rangle, \quad \text{(S17)}$$
$$H_\mu^\dagger(k)\left|u_{\mu,L}^{\pm}(k)\right\rangle = E_\pm^*(k)\left|u_{\mu,L}^{\pm}(k)\right\rangle, \quad \text{(S18)}$$

are not the same in general. Furthermore, one can develop the eigenvalue equation for the left eigenstate in (S18) by noticing that $H_\mu(k) = H_{\bar{\mu}}^\dagger(k)$ through (S12), where $\bar{\mu}$ labels the Hamiltonian block other than the μ-block, and that $E_\pm^*(k) = E_\pm(k)$ by the pseudo-Hermiticity condition, which imposes a real spectrum, leading to

$$H_{\bar{\mu}}(k)\left|u_{\mu,L}^{\pm}(k)\right\rangle = E_\pm(k)\left|u_{\mu,L}^{\pm}(k)\right\rangle, \quad \text{(S19)}$$

from where one can readily infer that

$$\left|u_{\mu,R}^{\pm}(k)\right\rangle = \left|u_{\bar{\mu},L}^{\pm}(k)\right\rangle, \quad \text{(S20)}$$

showing there to be a kind of "cross-talk" between the two pseudo-Hermitian blocks. For each block, its complete basis is formed by the *biorthogonal* basis [5], whose normalization condition for the eigenstates [6, 7] reads as

$$\left\langle u_{\mu,L}^{\sigma}(k)\middle|u_{\mu,R}^{\sigma'}(k)\right\rangle = \delta_{\sigma,\sigma'}, \quad \sigma, \sigma' = \pm, \quad \text{(S21)}$$

which, through (S20), can be rewritten as

$$\left\langle u_{\bar{\mu},R}^{\sigma}(k)\middle|u_{\mu,R}^{\sigma'}(k)\right\rangle = \delta_{\sigma,\sigma'}. \quad \text{(S22)}$$

By plugging (S15)-(S16) into (S22), and using (S2)-(S3), their respective normalization constants are found to obey the following relation,

$$\mathcal{N}_{2,R}^{\pm}(k)\mathcal{N}_{3,R}^{\pm}(k) = E_\pm(k). \quad \text{(S23)}$$

The extra condition that fixes the values of the normalization constants is derived by relating the problem back to the starting $\sqrt[3]{\text{SSH}}$ model, whose eigenstates can be grouped in three pairs of bands. One of these pairs has the form

$$\left|\psi_1^{\pm}(k)\right\rangle = \frac{1}{\sqrt{3}} \begin{pmatrix} \left|u_{\text{SSH}}^{\pm}(k)\right\rangle \\ \left|u_{2,R}^{\pm}(k)\right\rangle \\ \left|u_{3,R}^{\pm}(k)\right\rangle \end{pmatrix}, \tag{S24}$$

whose corresponding eigenvalue equation reads as

$$H_{\sqrt[3]{\text{SSH}}}(k) \left|\psi_1^{\pm}(k)\right\rangle = E_{\pm}^{\frac{1}{3}}(k) \left|\psi_1^{\pm}(k)\right\rangle, \tag{S25}$$

where the eigenvalues are directly obtained by taking the cubic-root of the eigenvalues of the cubed system in (S8). Then, by substituting (S15)-(S16) in (S24) and, in turn, inserting it in (S25), one finally obtains the values of the normalization constants,

$$\mathcal{N}_{2,R}^{\pm}(k) = E_{\pm}^{\frac{2}{3}}(k), \quad \mathcal{N}_{3,R}^{\pm}(k) = E_{\pm}^{\frac{1}{3}}(k), \tag{S26}$$

which agree with (S23), allowing us to rewrite (S24) as

$$\left|\psi_1^{\pm}(k)\right\rangle = \frac{1}{\sqrt{3}} \begin{pmatrix} \left|u_{\text{SSH}}^{\pm}(k)\right\rangle \\ E_{\pm}^{-\frac{2}{3}}(k) h_2 h_3 \left|u_{\text{SSH}}^{\pm}(k)\right\rangle \\ E_{\pm}^{-\frac{1}{3}}(k) h_3 \left|u_{\text{SSH}}^{\pm}(k)\right\rangle \end{pmatrix}. \tag{S27}$$

The other two pairs of eigenvalues and eigenstates can be obtained by making use of the generalized chiral symmetry of the system [1], as expressed in (S4). The eigenstates have the form

$$\left|\psi_2^{\pm}(k)\right\rangle = \Gamma_3 \left|\psi_1^{\pm}(k)\right\rangle = \frac{1}{\sqrt{3}} \begin{pmatrix} \left|u_{\text{SSH}}^{\pm}(k)\right\rangle \\ \omega_3 \left|u_{2,R}^{\pm}(k)\right\rangle \\ \omega_3^{-1} \left|u_{3,R}^{\pm}(k)\right\rangle \end{pmatrix}, \tag{S28}$$

$$\left|\psi_3^{\pm}(k)\right\rangle = \Gamma_3^2 \left|\psi_1^{\pm}(k)\right\rangle = \frac{1}{\sqrt{3}} \begin{pmatrix} \left|u_{\text{SSH}}^{\pm}(k)\right\rangle \\ \omega_3^{-1} \left|u_{2,R}^{\pm}(k)\right\rangle \\ \omega_3 \left|u_{3,R}^{\pm}(k)\right\rangle \end{pmatrix}, \tag{S29}$$

whose corresponding eigenvalue equations are then written as

$$H_{\sqrt[3]{\text{SSH}}}(k) \left|\psi_2^{\pm}(k)\right\rangle = \omega_3 E_{\pm}^{\frac{1}{3}}(k) \left|\psi_2^{\pm}(k)\right\rangle, \tag{S30}$$

$$H_{\sqrt[3]{\text{SSH}}}(k) \left|\psi_3^{\pm}(k)\right\rangle = \omega_3^{-1} E_{\pm}^{\frac{1}{3}}(k) \left|\psi_3^{\pm}(k)\right\rangle. \tag{S31}$$

As mentioned in Section 2 of the main text, the three-fold degenerate zero-energy point at $k = 0$ [see Fig. 2(a)] corresponds to an exceptional point of the spectrum [7], with only two associated eigenstates. One of them, $\left|\psi_1^{-}(0)\right\rangle = \frac{1}{\sqrt{2}}(1, -1, 0, 0, 0, 0)^T$, only has weight on the first sublattice, while the other, twice degenerate, $\left|\psi_2^{-}(0)\right\rangle = (t_1^{\frac{2}{3}} + t_2^{\frac{2}{3}})^{-\frac{1}{2}}(0, 0, \sqrt[3]{t_2}, -\sqrt[3]{t_1}, 0, 0)^T$, only has weight on the second sublattice.

As also stated in Section 2 of the main text, the bulk energy spectrum displays a ring energy gap for $\sqrt[3]{t_1} \neq \sqrt[3]{t_2}$, which is irreducible to the point or line gaps discussed in the literature [7, 8]. The explicit formula for the ring energy gap magnitude is given by

$$\Delta = E_R - E_r, \tag{S32}$$

where $E_R = \sqrt[3]{2t_{\max}}$ and $E_r = \sqrt[3]{2t_{\min}}$ are the outer and inner energy radii of the ring gap, respectively, with $t_{\max} = \max(t_1, t_2)$ and $t_{\min} = \min(t_1, t_2)$. This completes the analytical description of the bulk $\sqrt[3]{\text{SSH}}$ model.

From the form of $E_{\pm}(k)$ in (S8), it is straightforward to check, by considering the band limits occurring at the inversion-invariant momenta $k = 0, \pi$, that $E_{\pm}(k) \in \mathbb{R}_0^-$. In particular, we have $E_+(k) \in [-2t_{\min}, 0]$, as exemplified in

Fig. 2(d), which can be rewritten as $E_+(k) \in e^{i\pi}[0, 2t_{\min}]$. Then, by taking the cubic-root of the energy of this band, one obtains

$$E_+^{\frac{1}{3}}(k) \in e^{i\phi_+}[0, \sqrt[3]{2t_{\min}}], \quad \phi_+ = -\frac{\pi}{3}, \frac{\pi}{3}, \pi, \tag{S33}$$

with the same reasoning applying to the $E_-(k)$ band, that is, there is a $\phi_- = \phi_+$ phase factor appearing on the energies of the $\sqrt[3]{\text{SSH}}$ model due to the negative sign of the energies in the cubed SSH model. Due to the $\mathscr{C}_3$-symmetry of the model, one can choose any of the allowed $\phi_\pm$ in (S33), which basically selects one of the three energy branches [see examples of the three-branch structure in Figs. 2(a)-(c)], for the definition of $E_\pm^{\frac{1}{3}}(k)$ in (S25). The other energy branches are automatically obtained with (S30)-(S31).

Let us now perform the following Peierls substitution, $\sqrt[3]{t_1} \to \text{Exp}[i\frac{\pi}{3}(1+2q)]\sqrt[3]{t_1}$, with $q \in \mathbb{Z}$, in (S1). After cubing the Hamiltonian and diagonalizing the first diagonal block, relative to the parent SSH model, we get

$$E_\pm(k) = t_1 - t_2 \pm \sqrt{t_1^2 + t_2^2 - 2t_1 t_2 \cos k}. \tag{S34}$$

Comparing with (S8), we see that the sign of the last term of the argument of the square-root is flipped, amounting to a $k \to k + \pi$ sliding of both energy bands, and that t_1 in the constant energy shift coming from the identity proportional term in the Hamiltonian of (S7) now has a *positive* sign. As shown in Fig. 2(h) for $t_1 = 0.5$, the global energy shift of $2t_1$, in relation to Fig. 2(d), pushes one of the three-fold degenerate bands to the positive half of the spectrum. As a consequence, (S33) gets modified as

$$E_+^{\frac{1}{3}}(k) \in e^{i\phi_+}[0, \sqrt[3]{2t_{\min}}], \quad \phi_+ = -\frac{2\pi}{3}, 0, \frac{2\pi}{3}, \tag{S35}$$

while $\phi_- = -\frac{\pi}{3}, \frac{\pi}{3}, \pi$, since $E_-(k)$ is still in the negative part of the energy spectrum. Again, the choice of ϕ_+ and ϕ_- within the respective allowed sets is arbitrary since, after the choice is made, all other solutions are obtained by acting with Γ_3, as shown in (S28)-(S31). However, while for the previous case of zero Peierls phases in the hopping parameters one can choose $\phi_+ = \phi_-$ [which allows one to formally define the energy branches, as in Figs. 2(a)-(c)], now this is not possible since $\phi_+ \neq \phi_-$, regardless of how one chooses the phases. Therefore, one cannot define now energy branches, but only distinguish between the inner and outer sets of three energy bands that become degenerate upon cubing the spectrum, as illustrated by the color scheme in Figs. 2(e)-(g), where a $\frac{\pi}{3}$ relative phase shift can be seen between the sets, and the relevant ring gap appears now at $k = 0$ for $t_1 \neq t_2$.

Finally, we note that we do not consider general distributions of Peierls phases at the hopping terms outside the set defined above (S34). This is due to the fact that, for general distributions, one does not recover the purely real energy spectrum of an SSH model upon cubing the system, and we can no longer relate the features of the cubic-root model to a known topological and Hermitian parent model. We leave the study of n-root systems with non-Hermitian parent models for future studies.

A. Deviation from unidirectionality

The ring system of Fig. 1, with modulated gains and losses at the auxiliary rings, generates an imaginary gauge field incorporated in the effective hoppings between adjacent main rings through the h parameter as $t_\pm = te^{\pm h}$. From the Hamiltonian of the $\sqrt[3]{\text{SSH}}$ model in (S1), we parameterize the deviation from perfect unidirectionality as follows,

$$H'_{\sqrt[3]{\text{SSH}}}(k) = H_{\sqrt[3]{\text{SSH}}}(k) + \alpha H^\dagger_{\sqrt[3]{\text{SSH}}}(k), \tag{S36}$$

where $\alpha = e^{-2h}$. Assuming the same α parameterization under open boundary conditions (OBC), we show in Fig. S1 the energy spectrum of an $\sqrt[3]{\text{SSH}}$ chain with $N = 40$ unit cells as a function of the coupling ratio $\sqrt[3]{t_1}/\sqrt[3]{t_2}$, for different α values. As α is increased, little effect is observed on the real branch of the spectrum, except for some low-energy states. The two imaginary branches, in contrast, start bending more and more towards the real energy axis, eventually collapsing on it when Hermiticity is restored for $\alpha = 1$ in Fig. S1(e). From the system parameters indicated at the beginning of Section 3 in the main text, we obtain the same imaginary gauge field of $h = 2.07$ for both hopping terms $\sqrt[3]{t_1}$ and $\sqrt[3]{t_2}$, translating to $\alpha = 0.016$, which is very close to unidirectionality as can be seen, e.g., by comparing the numerical results of Fig. 3(a) with the analytical ones of Fig. 2(a). We note that even for deviations as large as $\alpha = 0.25$ [see Fig. S1(c)], the main qualitative features survive: (i) the presence of three distinct energy branches, except at the low-energy region of the spectrum, since the states near zero energy are the first ones to hybridize; (ii) the persistence of a ring gap away from the gap closing point $\sqrt[3]{t_1} = \sqrt[3]{t_2}$, harboring three in-gap edge states for the topological phase $\sqrt[3]{t_1} < \sqrt[3]{t_2}$.

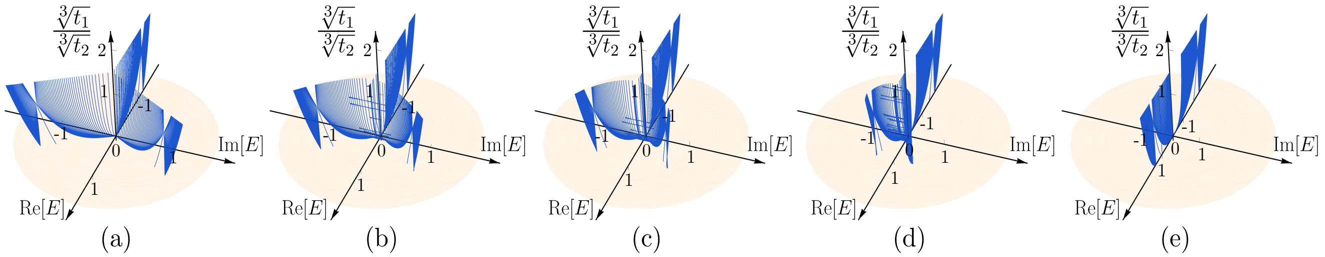

Figure S1. Complex energy spectrum, in units of $\sqrt[3]{t_2} \equiv 1$, as a function of the hopping ratio obtained from diagonalization of the Hamiltonian in (S36) for an open chain with $N = 40$ unit cells and (a) $\alpha = 0$, (b) $\alpha = 0.13$, (c) $\alpha = 0.25$, (d) $\alpha = 0.5$, and (e) $\alpha = 1$, where $\alpha \equiv t_-/t_+ = e^{-2h}$ is a global measure of the non-reciprocity of the hopping terms.

B. Symmetries

Let us now address the symmetries of the *full* $\sqrt[3]{\text{SSH}}$ model constructed with the photonic ring system depicted in Fig. 1. We recall that the main rings support states with opposite circulations $m = \pm 1$. The Hilbert space is enlarged as $\{|m, j(k)\rangle\} = \{|m\rangle\} \otimes \{|j(k)\rangle\}$, with $|j(k)\rangle$ defined above (S1). As can be understood from Fig. 1, a switch in the circulations at the main rings, $m = -1 \rightarrow m = 1$, amounts to a switch in the direction over which they connect with the losses and gains of the auxiliary rings (a connection through gains between main rings in one circulation becomes a connection through losses for the other circulation, and vice-versa). Consequently, all hopping directions are swapped when $m = -1 \rightarrow m = 1$, and the corresponding Hamiltonian becomes

$$H^{m=-1}_{\sqrt[3]{\text{SSH}}}(k) = H_{\sqrt[3]{\text{SSH}}}(k) \rightarrow H^{m=1}_{\sqrt[3]{\text{SSH}}}(k) = H^{\dagger}_{\sqrt[3]{\text{SSH}}}(k), \tag{S37}$$

and the total Hamiltonian, written in the enlarged Hilbert space, has the following block diagonal form

$$H_{\text{tot}} = \begin{pmatrix} H_{\sqrt[3]{\text{SSH}}}(k) & O_6 \\ O_6 & H^{\dagger}_{\sqrt[3]{\text{SSH}}}(k) \end{pmatrix}, \tag{S38}$$

where O_q is the zero square-matrix of size q. Recall that for the parameter values used in our numerical simulations, inter-circulation couplings, that is, those that couple the $m = 1$ and $m = -1$ circulations, can be safely neglected. For much smaller rings, or when using sharper transitions between regions with strong gain and loss, small off-diagonal blocks should be considered. In Table S1, we summarize the symmetries of $H_{\text{tot}}(k)$, indicating the matrix representation of their respective operators. Non-Hermitian symmetries [8] are labeled with the "†" symbol, with parity† symmetry the same as the pseudo-inversion symmetry defined in [9].

The y-mirror operation, M_y, performed about the central horizontal axis of the ring system in Fig. 1, acts as a y-reflection† operation, labeled R_y, on the position space,

$$R_y H_{\sqrt[3]{\text{SSH}}}(k) R_y^{-1} = H^{\dagger}_{\sqrt[3]{\text{SSH}}}(k), \tag{S39}$$

while simultaneously flipping the circulations in their space. Each of these space specific operations generates a global swap of all hopping directions, such that the two considered together act as a double negative that keeps the system invariant under M_y, as shown in Table S1.

The parity† symmetry involves an inversion operation within the position space, defined about the vertical axis crossing sites 3 and 5 in the unit cell at the bottom of Fig. 1,

$$P(k) H_{\sqrt[3]{\text{SSH}}}(k) P^{-1}(k) = H_{\sqrt[3]{\text{SSH}}}(-k). \tag{S40}$$

Note that the inversion operator $P(k)$, given explicitly in Table S1, is k-dependent, reflecting the fact that the inversion axis is shifted from the center of the unit cell. This has been shown to lead, in Hermitian systems, to nonquantized Zak's phases for the energy bands [10, 11]. When the complete ring system in Fig. 1 is considered, it is clear that the inversion operation switches the gain and loss regions of the link rings, leading to a global flip of the hopping directions, which is equivalent to a global flip of the circulations (an x Pauli matrix, τ_x, in the circulation space). As a consequence, the k-dependent parity† symmetry is obtained, whose operator $P_{\text{tot}}(k)$ and action on $H_{\text{tot}}(k)$ are given in Table S1.

We further define in Table S1 a flip† symmetry which acts non-trivially only in the circulation space, that is, the $m = \pm 1$ circulations are flipped under the action of its operator, $F = \tau_x \otimes I_6$. This can be understood as a global

Operator	Representation	Action on $H_{\rm tot}(k)$
y-reflection†	$R_y = \begin{pmatrix} \sigma_0 & 0 & 0 \\ 0 & 0 & \sigma_0 \\ 0 & \sigma_0 & 0 \end{pmatrix}$	n/a
Inversion	$P(k) = \begin{pmatrix} 0 & 1 & 0 & 0 & 0 & 0 \\ 1 & 0 & 0 & 0 & 0 & 0 \\ 0 & 0 & 1 & 0 & 0 & 0 \\ 0 & 0 & 0 & e^{-ik} & 0 & 0 \\ 0 & 0 & 0 & 0 & 1 & 0 \\ 0 & 0 & 0 & 0 & 0 & e^{-ik} \end{pmatrix}$	n/a
y-mirror	$M_y = \tau_x \otimes R_y$	$M_y H_{\rm tot}(k) M_y^{-1} = H_{\rm tot}(k)$
Parity†	$P_{\rm tot}(k) = \tau_x \otimes P(k)$	$P_{\rm tot}(k) H_{\rm tot}(k) P_{\rm tot}(k)^{-1} = H_{\rm tot}^\dagger(-k)$
Flip†	$F = \tau_x \otimes I_6$	$F H_{\rm tot}(k) F^{-1} = H_{\rm tot}^\dagger(k)$
Time-reversal†	$T = FK$	$T H_{\rm tot}(k) T^{-1} = H_{\rm tot}^\dagger(-k)$
Flip-time	$FT = (\tau_0 \otimes I_6)\mathrm{K}$	$FT H_{\rm tot}(k) T^{-1} F^{-1} = H_{\rm tot}(-k)$
Flip-inversion	$FP_{\rm tot}(k) = \tau_0 \otimes P(k)$	$FP_{\rm tot}(k) H_{\rm tot}(k) P_{\rm tot}(k)^{-1} F^{-1} = H_{\rm tot}(-k)$
Parity-time	$P_{\rm tot}(k)T = \big(\tau_0 \otimes P(k)\big)K$	$P_{\rm tot}(k) T H_{\rm tot}(k) T^{-1} P_{\rm tot}(k)^{-1} = H_{\rm tot}(k)$
Generalized Chiral	$\Gamma_3^{\rm tot} = \Gamma_3 \oplus \Gamma_3^\dagger$	$\Gamma_3^{\rm tot} H_{\rm tot}(k) {\Gamma_3^{\rm tot}}^{-1} = \omega_3^{-1} H_{\rm tot}(k)$
Generalized Particle-hole	$S_3^{\rm tot} = \Gamma_3^{\rm tot} FT$	$S_3^{\rm tot} H_{\rm tot}(k) {S_3^{\rm tot}}^{-1} = \omega_3^{-1} H_{\rm tot}(-k)$

Table S1. Operators and symmetries of $H_{\rm tot}(k)$ in (S38), with the exception of the y-reflection† and inversion, which are operations acting only on the position space, defined as $\{|j(k)\rangle\}$, with $j = 1, 2, \ldots, 6$ labeling the corresponding site within the unit cell. τ_0 (τ_x) is the 2×2 identity matrix (x Pauli matrix) acting on the space of circulations. Γ_3 is given in (S5) and $\omega_3 = e^{i\frac{2\pi}{3}}$.

reversal of the hopping directions, such that it can also be viewed, in terms of the ring system of Fig. 1, as a global swap of the gain and loss regions within each link ring.

Some care is needed for deriving the time-reversal operator. It is defined as an anti-unitary operator of the form $T = UK$, where K is the complex conjugation operator obeying $KO = O^*K$ and $KK^{-1} = I$, while U is a unitary operator that, for a general angular momentum, and up to a global phase factor, can be written as

$$U = -e^{-i\pi J_y/\hbar}, \tag{S41}$$

where $\hbar$ is the reduced Planck's constant and J_y is the y-component of the angular momentum operator. Assuming a bosonic pseudospin-1 system, the basis spans $\{|1,1\rangle = (1,0,0)^T, |1,0\rangle = (0,1,0)^T, |1,-1\rangle = (0,0,1)^T\}$, where the states are written in the form $|l,m\rangle$, and one has

$$J_y = \frac{i\hbar}{\sqrt{2}} \begin{pmatrix} 0 & -1 & 0 \\ 1 & 0 & -1 \\ 0 & 1 & 0 \end{pmatrix}, \tag{S42}$$

$$U = \begin{pmatrix} 0 & 0 & 1 \\ 0 & -1 & 0 \\ 1 & 0 & 0 \end{pmatrix}. \tag{S43}$$

It is clear that $U|l,m\rangle = (-1)^{m+1}|l,-m\rangle$, *i.e.*, that U reverses the circulations. In our ring system, the accessible states are the ones with opposite circulations, $m = \pm 1$. The $m = 0$ circulation does not correspond to any propagating resonant mode in the ring system and, as such, can be dropped to get, in the $\{|1,1\rangle, |1,-1\rangle\}$ subspace, $U = \tau_x$. When the entire Hilbert space is considered, one can define an operator that flips circulations as $F = \tau_x \otimes I_6$ and, from it, the time-reversal operator as $T = FK$ (see Table S1). Under the time-reversal operation, and apart from the usual conjugation operation, one flips the circulations. It should also be noted that $T^2 = 1$, consistent with the bosonic nature of our system. This also explains why we had to start from the J_y matrix of a pseudospin-1 particle in (S42), and then reduce it to the relevant subspace of $m = \pm 1$ circulations, rather than considering the J_y matrix of a two-state pseudospin-$\frac{1}{2}$ particle, which would lead to $T^2 = -1$. Table S1 shows that the total system has the non-Hermitian time-reversal† symmetry. When it is combined with the flip operation (which duplicates the one already contained in $T = FK$), we obtain a new kind of symmetry for the full model, which we label *flip-time symmetry*, whose operator is given by the product FT.

The generalized chiral symmetry $\mathscr{C}_3$ is also present in the full model of (S38), and its operator, $\Gamma_3^{\rm tot}$, is given by the direct sum of the $\mathscr{C}_3$ symmetry operators of each circulation (see Table S1), with each being the Hermitian

conjugated version of the other. Finally, we also define in Table S1 a generalized particle-hole symmetry [1], whose operator can be written as a product of three operators, $S_3^{\text{tot}} = \Gamma_3^{\text{tot}} FT$. Conversely, note that the operator of the generalized chiral symmetry, in the full system, is not obtained in the usual way, that is, as the product of generalized particle-hole and time-reversal symmetries, $\Gamma_3^{\text{tot}} \neq S_3^{\text{tot}} T$, but rather as the product of generalized particle-hole and flip-time symmetries, $\Gamma_3^{\text{tot}} = S_3^{\text{tot}} FT$.

II. HIGHER-ORDER ROOTS

In Fig. 4, we depict the unit cell of the n-root SSH ($\sqrt[n]{\text{SSH}}$) model, which is composed of $2n$ sites, connected through unidirectional couplings, and n two-site sublattices. Under periodic boundary conditions (PBC), the bulk Hamiltonian of this model, in the ordered $\{|j(k)\rangle\}$ basis, with $j = 1, 2, \ldots, 2n$ (see Fig. 4), generalizes (S1) as

$$H_{\sqrt[n]{\text{SSH}}}(k) = \begin{pmatrix} & h_1 & & & \\ & & h_2 & & \\ & & & \ddots & \\ & & & & h_{n-1} \\ h_n & & & & \end{pmatrix}, \tag{S44}$$

$$h_1 = h_n^\dagger = -\begin{pmatrix} \sqrt[n]{t_1'} & \sqrt[n]{t_2'} e^{-ik} \\ \sqrt[n]{t_1'} & \sqrt[n]{t_2'} \end{pmatrix}, \tag{S45}$$

$$h_l = -\begin{pmatrix} \sqrt[n]{t_1} & 0 \\ 0 & \sqrt[n]{t_2} \end{pmatrix}, \quad l = 2, 3, \ldots, n-1, \tag{S46}$$

where the entries not shown are zeros and the lattice spacing is set to unity. This constitutes a particular realization of the quasi-1D n-partite models studied in [1], and therefore this Hamiltonian obeys a generalized chiral symmetry,

$$\mathscr{C}_n: \ \Gamma_n H_{\sqrt[n]{\text{SSH}}}(k) \Gamma_n^{-1} = \omega_n^{-1} H_{\sqrt[n]{\text{SSH}}}(k), \tag{S47}$$

$$\Gamma_n = \text{diag}(\sigma_0, \omega_n \sigma_0, \omega_n^2 \sigma_0, \ldots, \omega_n^{n-1} \sigma_0), \tag{S48}$$

with $\omega_n = e^{i\frac{2\pi}{n}}$. In the language introduced by some of the authors in [1], the set

$$\{H_{\sqrt[n]{\text{SSH}}}(k), \omega_n^{-1} H_{\sqrt[n]{\text{SSH}}}(k), \omega_n^{-2} H_{\sqrt[n]{\text{SSH}}}(k), \ldots, \omega_n H_{\sqrt[n]{\text{SSH}}}(k)\} \tag{S49}$$

spans the n chiral colors of the same Hamiltonian, and any color can be turned into any other by applying the $\mathscr{C}_n$-symmetry operation in (S47) a given amount of times. After raising the Hamiltonian in (S44) to the nth-power we obtain the following block diagonal matrix, with each block defined in a single sublattice,

$$H^n_{\sqrt[n]{\text{SSH}}}(k) = \text{diag}(H_{\text{SSH}'}(k), H_2(k), \ldots, H_n(k)), \tag{S50}$$

where the first block is an energy shifted SSH Hamiltonian,

$$\begin{aligned} H_{\text{SSH}'}(k) &= h_1 h_2 \ldots h_n \\ &= -\begin{pmatrix} w_1 + w_2 & w_1 + w_2 e^{-ik} \\ w_1 + w_2 e^{ik} & w_1 + w_2 \end{pmatrix} \\ &= -(w_1 + w_2)\sigma_0 + H_{\text{SSH}}(k), \end{aligned} \tag{S51}$$

with renormalized hopping parameters, $w_i := t_i'^{\frac{2}{n}} t_i^{\frac{n-2}{n}}$ for n odd and $w_i \to -w_i$ for n even, due to the sign convention for the hopping terms in (S45)-(S46). Its eigenvalues and eigenstates are given in (S8)-(S9), with $t_i \to w_i$. The other diagonal blocks of (S50) are given by

$$H_j(k) = h_j h_{j+1} \ldots h_{n-1+j}, \quad j = 2, 3, \ldots, n, \tag{S52}$$

respecting the periodic condition $j = n+1 \to j = 1$. All these blocks are isospectral to $H_{\text{SSH}'}(k)$ [1] and obey the relation $H_j = H^\dagger_{n+2-j}$ (the k dependence is omitted henceforth), such that they are pseudo-Hermitian matrices, except when $j = \frac{n}{2} + 1$ for n even, which yields a Hermitian block $H_{\frac{n}{2}+1} = H^\dagger_{\frac{n}{2}+1}$. The eigenvalue equation for the right and left eigenstates of each of these blocks is written as

$$H_j \left| u^\pm_{j,R}(k) \right\rangle = E_\pm(k) \left| u^\pm_{j,R}(k) \right\rangle, \tag{S53}$$

$$H_j^\dagger \left| u^\pm_{j,L}(k) \right\rangle = H_{n+2-j} \left| u^\pm_{j,L}(k) \right\rangle = E_\pm(k) \left| u^\pm_{j,L}(k) \right\rangle, \tag{S54}$$

from where it is clear that $\left|u^{\pm}_{j,L}(k)\right\rangle = \left|u^{\pm}_{n+2-j,R}(k)\right\rangle$, and with

$$\left|u^{\pm}_{j,R}(k)\right\rangle = E_{\pm}^{-\frac{n+1-j}{n}}(k) h_j h_{j+1} \dots h_n \left|u^{\pm}_{\mathrm{SSH}}(k)\right\rangle, \tag{S55}$$

where a finite energy, $|E_{\pm}(k)| > 0$, is assumed.

The relations above allow us to go back and determine the analytical solutions of the starting $\sqrt[n]{\mathrm{SSH}}$ model. Similarly to (S25), we determine all solutions from the eigenvalue equation for branch 1,

$$H_{\sqrt[n]{\mathrm{SSH}}}(k) \left|\psi^{\pm}_1(k)\right\rangle = E_{\pm}^{\frac{1}{n}}(k) \left|\psi^{\pm}_1(k)\right\rangle, \tag{S56}$$

with

$$\left|\psi^{\pm}_1(k)\right\rangle = \frac{1}{\sqrt{n}} \begin{pmatrix} \left|u^{\pm}_{\mathrm{SSH}}(k)\right\rangle \\ \left|u^{\pm}_{2,R}(k)\right\rangle \\ \left|u^{\pm}_{3,R}(k)\right\rangle \\ \vdots \\ \left|u^{\pm}_{n,R}(k)\right\rangle \end{pmatrix}, \tag{S57}$$

which can be rewritten, through (S55), as

$$\left|\psi^{\pm}_1(k)\right\rangle = \frac{1}{\sqrt{n}} \begin{pmatrix} \left|u^{\pm}_{\mathrm{SSH}}(k)\right\rangle \\ E_{\pm}^{-\frac{n-1}{n}}(k) h_2 h_3 \dots h_n \left|u^{\pm}_{\mathrm{SSH}}(k)\right\rangle \\ E_{\pm}^{-\frac{n-2}{n}}(k) h_3 \dots h_n \left|u^{\pm}_{\mathrm{SSH}}(k)\right\rangle \\ \vdots \\ E_{\pm}^{-\frac{1}{n}}(k) h_n \left|u^{\pm}_{\mathrm{SSH}}(k)\right\rangle \end{pmatrix}. \tag{S58}$$

The energy can be found by generalizing (S33) as $E_{+}^{\frac{1}{n}}(k) \in e^{i\phi_+}[0, \sqrt[n]{2w_{\mathrm{min}}}]$, with $w_{\mathrm{min}} = \min(w_1, w_2)$ and

$$\begin{cases} \phi_+ = \frac{\pi}{n}(1+2l) \pmod{2\pi}, \quad l = 0, 1, \dots, n-1, \; n \text{ odd}, \\ \phi_+ = \frac{2\pi}{n} l \pmod{2\pi}, \quad l = 0, 1, \dots, n-1, \; n \text{ even}, \end{cases} \tag{S59}$$

with $\phi_- = \phi_+$. As before, one can choose any $\phi_{\pm}$ from the allowed set, that is, any l in (S59), to define the complex energy phase of branch 1 in (S56) and of the respective eigenstate components in (S58). After setting $\phi_{\pm}$ as one of the possible values in (S59), the other branches are determined from successive applications of Γ_n in (S47) as

$$\left|\psi^{\pm}_j(k)\right\rangle = \Gamma_n^{j-1} \left|\psi^{\pm}_1(k)\right\rangle = \frac{1}{\sqrt{n}} \begin{pmatrix} \left|u^{\pm}_{\mathrm{SSH}}(k)\right\rangle \\ \omega_n^{j-1} \left|u^{\pm}_{2,R}(k)\right\rangle \\ \omega_n^{2(j-1)} \left|u^{\pm}_{3,R}(k)\right\rangle \\ \vdots \\ \omega_n^{-(j-1)} \left|u^{\pm}_{n,R}(k)\right\rangle \end{pmatrix}, \tag{S60}$$

again with $j = 2, 3 \dots, n$, and leading to

$$H_{\sqrt[n]{\mathrm{SSH}}}(k) \left|\psi^{\pm}_j(k)\right\rangle = \omega_n^{j-1} E_{\pm}^{\frac{1}{n}}(k) \left|\psi^{\pm}_j(k)\right\rangle. \tag{S61}$$

As we have seen before for the $\sqrt[3]{\mathrm{SSH}}$ model, there will be now an n-fold degenerate zero-energy point at $k = 0$ that corresponds to an exceptional point of the spectrum, with the same two associated eigenstates. One of them, $\left|\psi^{-}_1(0)\right\rangle = \frac{1}{\sqrt{2}}(1, -1, 0, 0, \vec{O}_{2n-4})^T$, with $\vec{O}_{2n-4}$ a zero vector of size $2n-4$, only has weight on the first sublattice, while the other, $(n-1)$-fold degenerate, $\left|\psi^{-}_2(0)\right\rangle = (t_1^{\frac{2}{n}} + t_2^{\frac{2}{n}})^{-\frac{1}{2}}(0, 0, \sqrt[n]{t_2}, -\sqrt[n]{t_1}, \vec{O}_{2n-4})^T$, only has weight on the

second sublattice, since the rungs in Fig. 4 effectively behave, for this eigenstate, as unidirectional Hatano-Nelson chains [12] with all the amplitude accumulated at the edge rung sites 3 and 4.

In Fig. S2, we show the bulk energy spectrum of a very high root system, namely the $\sqrt[20]{\text{SSH}}$ model, for $\sqrt[20]{t_1} = 0.6$ and $\sqrt[20]{t_2} = 1$. Twenty two-band branches, separated by $\frac{\pi}{10}$ angular increments in the energy plane, are present there. The ring gap between the bottom of the outer bands and the top of the inner bands, occurring at $k = \pi$, becomes more evident as n increases, becoming continuous in the $n \to \infty$ limit.

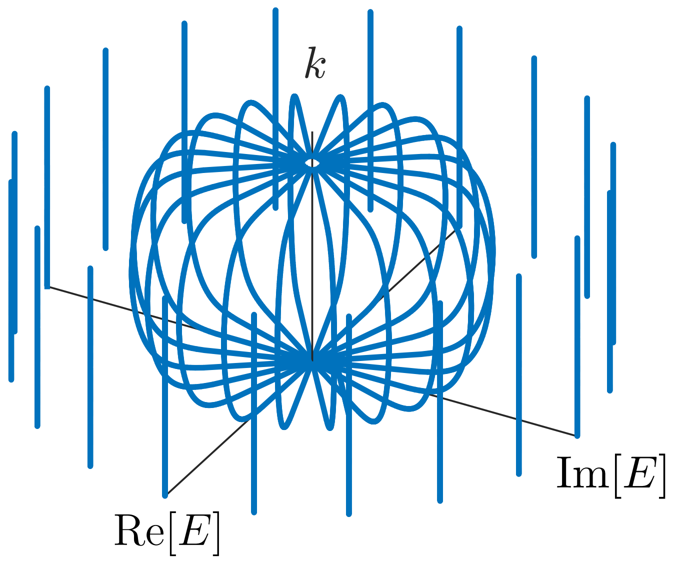

Figure S2. Complex bulk energy spectrum, in units of $\sqrt[20]{t_2} = 1$, of the $\sqrt[20]{\text{SSH}}$ model, with $\sqrt[20]{t_1} = 0.6$

Finally, if the Peierls substitution $\sqrt[n]{t_i^{(\prime)}} \to \sqrt[n]{t_i^{(\prime)}} e^{i\frac{\pi}{n}}$ is applied to $i = 1 \vee 2$, then a π flux is generated within the triangles of the plaquette with $\sqrt[n]{t_i^{(\prime)}}$ couplings in Fig. 4. This causes a global sign shift in one of the $E_\pm(k)$ bands in H'_{SSH}, relative to the other, which, as for the cubic-root model in Fig. 2(e)-(g), causes a $\frac{\pi}{n}$ shift between inner and outer branches of the energy spectrum. We label this system as the $\sqrt[n]{\text{SSH}}_{\frac{\pi}{n}}$ model. Below, we will show an example of this shift when we analyze the $n = 5$ case in its photonic ring realization. We point out that the $\sqrt[n]{\text{SSH}}$ and $\sqrt[n]{\text{SSH}}_{\frac{\pi}{n}}$ models are topologically distinct, in the sense that one cannot continuously interpolate between the two without breaking at least the $\mathscr{C}_n$-symmetry of the system.

It should also be stressed that our scheme of generating $\sqrt[n]{\text{TIs}}$ is fundamentally different from that recently proposed in [13]. There, the n-root of a lattice is obtained by a direct extension of the split graph [14] structure of several $\sqrt{\text{TIs}}$ [15–20]. Namely, each link/hopping of the TI is subdivided into n equal parts by adding $n-1$ sites between the two original ones. The resulting model is always bipartite, regardless of n. Upon raising its Hamiltonian to the nth-power, the parent TI and topologically equivalent models are obtained as diagonal blocks. However, a global off-diagonal term, labeled enhanced Hamiltonian, couples the diagonal blocks with each other in non-trivial ways, and it is not yet clear how this mixing ultimately affects the clean topological characterization of the diagonal blocks. Our scheme for constructing $\sqrt[n]{\text{TIs}}$, on the other hand, relies on substituting the links of the parent TI with loop modules composed of unidirectional links. The resulting system is automatically n-partite, according to the definition of [1], and therefore has a built-in generalized chiral symmetry $\mathscr{C}_n$. Furthermore, when the Hamiltonian is raised to the nth-power, all off-diagonal blocks vanish, and a direct hierarchical relation, free of ambiguity, can be established between the $\sqrt[n]{\text{TI}}$ and the parent TI.

A. Five-root model

To demonstrate that models with a higher root degree than the ones showcased in the main text are implementable, we build the $\sqrt[5]{\text{SSH}}$ model with the unit cell displayed in Fig. S3(a). For this case, the long link ring has semiaxis lengths of $R_{a1} = 10.74\,\mu\text{m}$ and $R_{b1} = 2.5\,\mu\text{m}$ and a maximum loss value of $\text{Im}(\tilde{n}_{\text{link}}) = 0.058$, whereas the short rings are the same as the ones used in Section 4 in the main text. In Fig. S3(b)-(e), the same pattern of results as in the main text is repeated, now showcasing the five-fold splitting characteristic of a five-partite system. In principle,

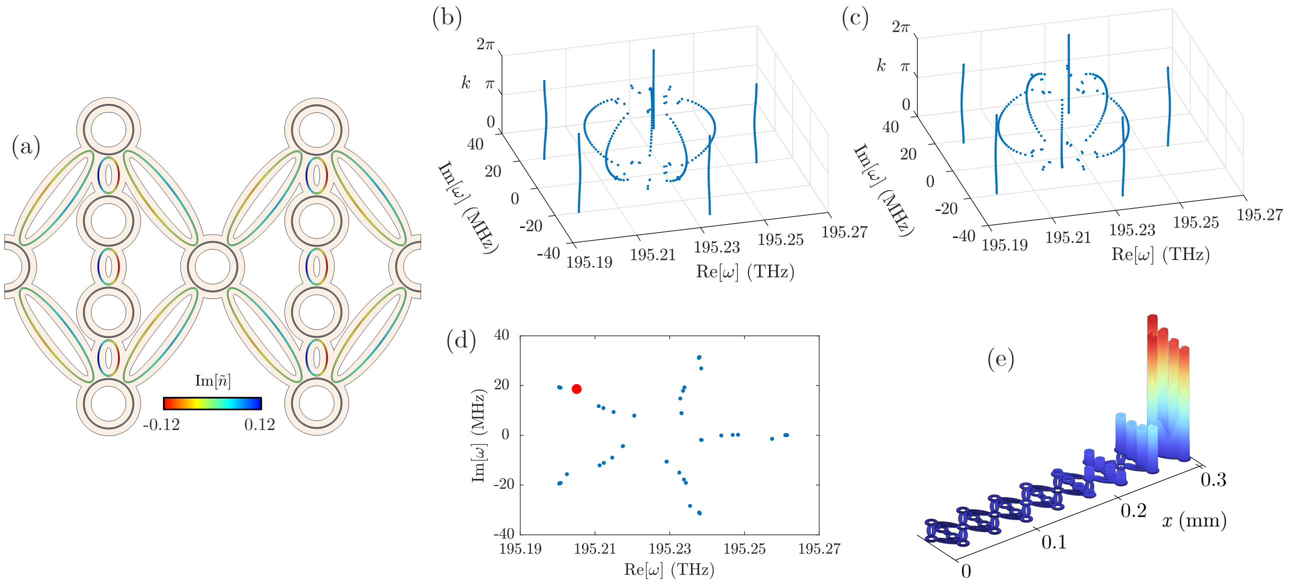

Figure S3. (a) Unit cell for the $\sqrt[5]{\text{SSH}}$ model. Shorther (longer) link resonators display stronger (weaker) gain and loss values. Eigenfrequencies of the photonic (b) $\sqrt[5]{\text{SSH}}$ model and (c) $\sqrt[5]{\text{SSH}}_{\frac{\pi}{5}}$ model with PBC at steps of $\Delta k = 0.05\pi$. (d) Eigenfrequencies of the photonic $\sqrt[5]{\text{SSH}}$ chain with OBC and $N = 4$ unit cells, for $d_1 = 0.33\,\mu\text{m}$ and $d_2 = 0.3\,\mu\text{m}$, where the five-fold splitting of the bands can be readily observed. (e) Electric field norms for the edge mode of the system indicated by the red point in (d).

any n-root model can be constructed in this manner, at the cost of increasing the system size. Comparing these figures with Fig. 3 and Fig. 5 in the main text, corresponding to the cubic- and quartic-root models, one might note some deviations, particularly in the edge modes of Fig. S3(d) which are slightly displaced from their branches. This is mainly caused by the elongated nature of the link rings with sharp bends, which are inherently lossier than the circular rings. By virtue of the geometrical constraints, the ellipticity in this case is made large enough for distortions to appear in the spectrum. Although these are expected to increase for higher-order roots, one may move away from the elliptic design and use longer links with rounder edges to mitigate these effects.

III. POLARIZATION

In a non-Hermitian system governed by a Hamiltonian H of dimension N, the right (R) and left (L) eigenstates, defined by

$$H\left|\psi_l^R\right\rangle = E_l\left|\psi_l^R\right\rangle,$$
$$H^\dagger\left|\psi_l^L\right\rangle = E_l^*\left|\psi_l^L\right\rangle, \tag{S62}$$

where $l = 1, 2, \ldots, N$ is the site index, assumed ordered in $|E_l|$, are different in general. A biorthogonal basis [7], which is complete if exceptional points are absent from the system, can be constructed from the normalization condition $\left\langle\psi_l^L\middle|\psi_m^R\right\rangle = \delta_{lm}$. For a generic operator $\hat{O}$, its biorthogonal expectation value is given by

$$\left\langle\psi_l^L\right|\hat{O}\left|\psi_l^R\right\rangle = O_l \in \mathbb{C}. \tag{S63}$$

Our goal is to define a polarization operator for the non-Hermitian $\sqrt[n]{\text{SSH}}$ model. The polarization of Hermitian models relies on the existence of a Fermi level E_F that identifies the occupied states contributing to it. However, the concept of a Fermi level is a foreign one for a non-Hermitian model with a complex energy spectrum, and therefore there is no general method for defining the set of states that are to be considered occupied in order to compute the polarization. On the other hand, by taking advantage of the ring energy gap of the $\sqrt[n]{\text{SSH}}$ model, and in analogy with Hermitian systems, we define a *ring Fermi level* of radius $|E_F|$, as exemplified in Fig. 3(a), such that all states within the ring are considered occupied, while the ones outside it are taken to be unoccupied. In this way, the polarization

operator is written as

$$\hat{\mathcal{P}} = \frac{e}{N}\sum_{j=1}^{N} j\hat{\Pi}_j, \tag{S64}$$

$$\hat{\Pi}_j = \sum_{\alpha=1}^{2n} |j,\alpha\rangle\langle j,\alpha|, \tag{S65}$$

where e is the electron charge, N the number of unit cells, $2n$ is the number of sites per unit cell of the $\sqrt[n]{\text{SSH}}$ model, and $|j,\alpha\rangle$ is the basis state at site α of unit cell j. For a given filling factor, which is set by the ring Fermi level, the biorthogonal polarization of an $\sqrt[n]{\text{SSH}}$ chain is defined as

$$\mathcal{P} := \sum_{l=1}^{l_F} \langle\psi_l^L|\,\hat{\mathcal{P}}\,|\psi_l^R\rangle, \tag{S66}$$

where l_F is the index of the eigenstate with highest $|E| \leq |E_F|$, and corresponds to $l_F = nN$ at half-filling.

As explained is the main text, due to the "particle-hole duality" between the original $\sqrt[n]{\text{SSH}}$ and parent SSH models, wherein the occupied states on one side correspond to the unoccupied states on the other, and vice-versa, we are instead interested, for comparison purposes, in the complementary half-filled condition, where the sum limits in (S66) are inverted as $\sum_{l=1}^{nN} \to \sum_{l=nN+1}^{2nN}$, which is ultimately equivalent to performing $\mathcal{P} \to -\mathcal{P}$. For $n = 3$, this leads to the polarization formula in (9) of the main text.

We view the biorthogonal polarization defined in (S66) as a natural extension of the usual polarization (defined for Hermitian systems such as the parent SSH model, where left and right eigenstates are equivalent) to the non-Hermitian $\sqrt[n]{\text{SSH}}$ models studied here, thereby enabling a direct comparison between the polarizations of the root and parent models. To further highlight the physical meaning of $\mathcal{P}$, we depict in Fig. S4 an open $\sqrt[3]{\text{SSH}}$ chain at half-filling,

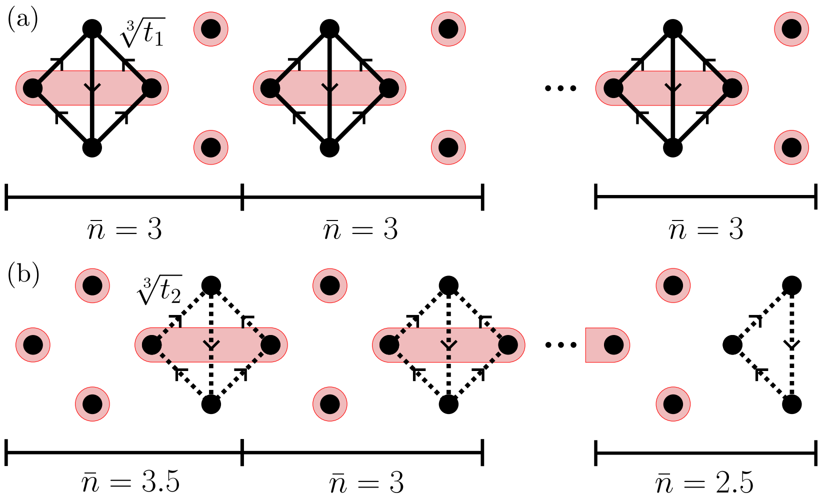

Figure S4. Illustration of an open $\sqrt[3]{\text{SSH}}$ chain at half-filling (filling the states within the ring Fermi level) in the atomic limit (a) $t_1 > 0$ and $t_2 = 0$, and (b) $t_1 = 0$ and $t_2 > 0$. The occupied states are colored in red and all have zero energy at these limits, with the ones spanning two sites corresponding to antisymmetric linear combinations with the same weight at each site. The average number of particles $\bar{n}$ at each unit cell is indicated at the bottom of each plot.

considering the two opposite atomic limits. When $t_2 = 0$, in Fig. S4(a), the particle density is balanced for all unit cells, such that $\mathcal{P} = 0$. When $t_1 = 0$, in Fig. S4(b), on the other hand, there is an excess of one particle at the first unit cell, in relation to the last one, which leads to $\mathcal{P} = -\frac{e}{2}$. These quantized polarization values fully agree with the corresponding limits of the polarization curve of Fig. 3(c), where one finds $\mathcal{P} = 0$ for $t_2 = 0$ and $\mathcal{P} \to \frac{e}{2}$ for $t_2 \to \infty$ (remember we are ploting there the complementary half-filling condition, implying $\mathcal{P} \to -\mathcal{P}$).

Finally, we note that the biorthogonal polarization, as defined in (S66), should be distinguished from the one formulated in recent works [6, 21]. There, the polarization is computed over the set of edge states, and therefore an *a priori* knowledge, whether analytical or numerical, of the boundary modes is required. No such knowledge is assumed

in our formulation, which is agnostic with regards to the precise nature of the occupied states that contribute to the polarization. Furthermore, the formulation in Refs. [6, 21] focuses on strictly zero-energy edge states originated from sublattice imbalance in bipartite models, whereas in our case the biorthogonal polarization is computed for any model with a well defined ring Fermi level, such as the n-partite [1] $\sqrt[n]{\text{SSH}}$ models studied here, and implies no constraint on the energy of the edge states. In fact, the ones appearing throughout this work all have finite energies. For these reasons, the polarization in (S66) comes as a natural generalization of the polarization of Hermitian systems to non-Hermitian models with biorthogonal bases.

IV. PROOF OF NONRECIPROCITY

As sketched in Fig. S5, we consider a set of main rings of radius R_M coupled through an antiresonant link ring of radius R_L with nonreciprocity parameter h, which is displaced a small distance y from the line between the center of the two neighboring main rings so that each arm picks up a phase factor of ϕ. All rings have a width of w and we consider a separation of d between their outer radii. We follow the derivation detailed in Ref. [22], which relates the field amplitudes in neighboring rings using the transfer matrix formulation to obtain the following relations,

$$\begin{pmatrix} E_c \\ E_d \end{pmatrix} = \frac{1}{i\kappa}\begin{pmatrix} \tau & -1 \\ 1 & -\tau \end{pmatrix}\begin{pmatrix} E_b \\ E_a \end{pmatrix} = M_1 \begin{pmatrix} E_b \\ E_a \end{pmatrix}, \tag{S67}$$

$$\begin{pmatrix} E_f \\ E_e \end{pmatrix} = \begin{pmatrix} e^{i(\beta L_L/2+\phi)}e^h & 0 \\ 0 & e^{i(-\beta L_L/2+\phi)}e^h \end{pmatrix}\begin{pmatrix} E_c \\ E_d \end{pmatrix} = M_2 \begin{pmatrix} E_c \\ E_d \end{pmatrix}, \tag{S68}$$

$$\begin{pmatrix} E_g \\ E_h \end{pmatrix} = \frac{-1}{i\kappa}\begin{pmatrix} \tau & -1 \\ 1 & -\tau \end{pmatrix}\begin{pmatrix} E_f \\ E_e \end{pmatrix} = M_3 \begin{pmatrix} E_f \\ E_e \end{pmatrix}, \tag{S69}$$

$$\begin{pmatrix} E_j \\ E_i \end{pmatrix} = \begin{pmatrix} e^{-i\beta L_M/2} & 0 \\ 0 & e^{i\beta L_M/2} \end{pmatrix}\begin{pmatrix} E_g \\ E_h \end{pmatrix} = M_4 \begin{pmatrix} E_g \\ E_h \end{pmatrix}, \tag{S70}$$

where τ and κ are the transmission and coupling coefficients, β is the propagation constant in the rings, $L_M = 2\pi[R_M + w/2]$ (L_L) is the circumference length of the main (link) rings and E_l are the field amplitudes as labelled in Fig. S5. For a periodic system where we can apply Bloch's theorem,

$$\begin{pmatrix} E_j \\ E_i \end{pmatrix} = e^{ik}\begin{pmatrix} E_b \\ E_a \end{pmatrix}, \tag{S71}$$

where the lattice spacing is set to $a \equiv 1$. We can obtain a solution by imposing $|M_4M_3M_2M_1 - e^{ik}I| = 0$, with I the identity matrix. Close to resonance of the main rings, we can apply the following substitution: $\sin(\beta L_M) \approx (\omega - \omega_0)L_M/v_g$, where ω_0 is the resonant frequency and v_g the group velocity [22, 23]. Using this approximation, in addition to the antiresonant condition for the link rings, results in the following solution for the dispersion relation:

$$\omega(k) = \omega_0 + t\left[e^h e^{i(\phi-k)} + e^{-h}e^{-i(\phi-k)}\right], \tag{S72}$$

where $t = v_g\kappa^2/L_M$ is the coupling strength between main rings. This dispersion relation is exactly the same as the one for a system with asymmetric coupling $t_\pm = te^{\pm h}e^{\pm i\phi}$, thus proving that the considered ring setup can generate such a coupling.

V. EXTRACTING THE COUPLING BETWEEN RINGS

We consider that the main rings (MRs) have a resonance frequency ω_0 and that they are coupled through a link ring (LR) that creates an effective asymmetric coupling. If we focus on the basic MR-LR-MR block of Fig. S5, the Hamiltonian is given by

$$H = \begin{pmatrix} \omega_0 & te^h \\ te^{-h} & \omega_0 \end{pmatrix}, \tag{S73}$$

whose eigenvalues are $\omega_\pm = \omega_0 \pm t$. Therefore, by numerically obtaining the eigenvalues of the basic 3-ring block, we can obtain the coupling between main rings by computing

$$t = \frac{1}{2}(\omega_+ - \omega_-). \tag{S74}$$

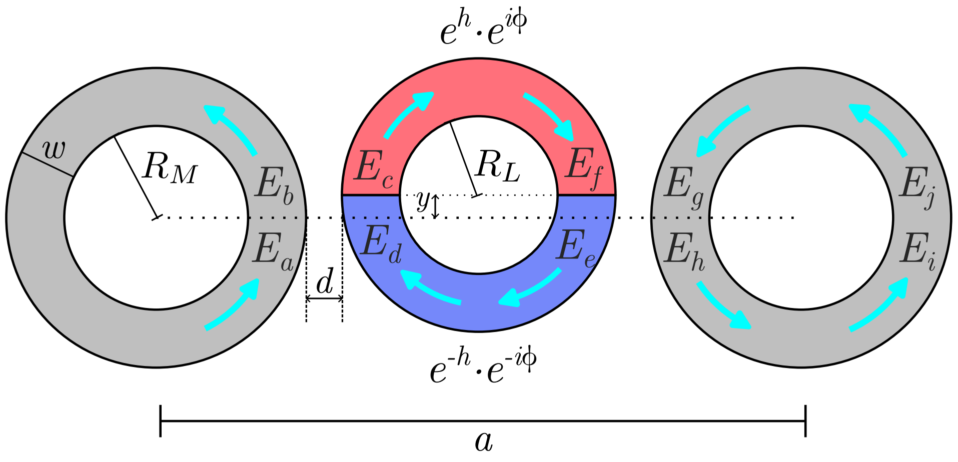

Figure S5. Sketch of a set of two main rings in grey, coupled through a link ring with a split gain and loss distribution, which yields a $e^{\pm h}$ term in the coupling depending on the direction. The blue arrows indicate the circulations and the field amplitudes that are coupled in each ring. A vertical displacement from the coupling line leads to a real phase ϕ in the coupling.

In Fig. S6(a) and (b), we show the eigenvalues of the basic ring block and the coupling computed from them as a function of the distance between main and link rings, respectively.

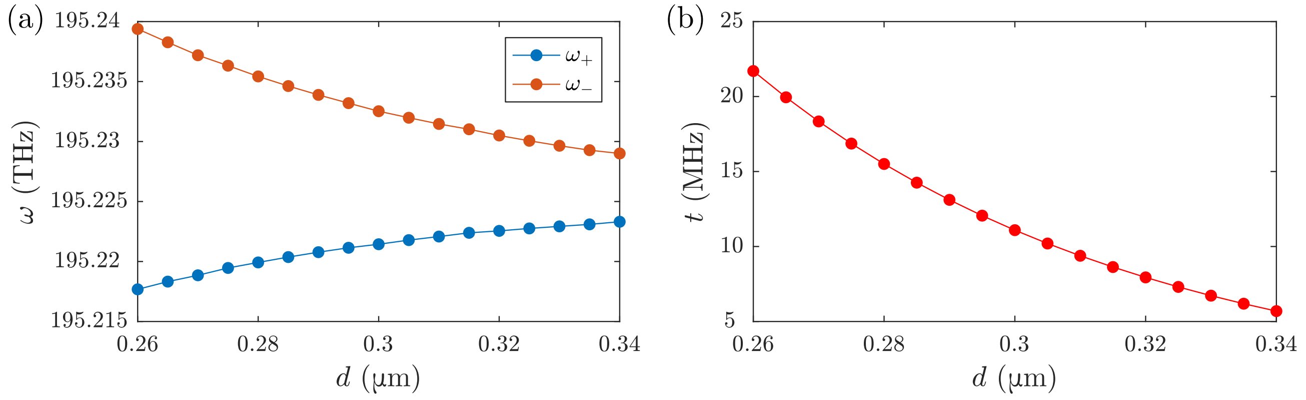

Figure S6. (a) Eigenfrequencies of the set depicted in Fig. S5, composed of two main rings coupled through a link ring, and (b) coupling between main rings computed through (S74) using the eigenfrequencies in (a), with respect to the relative distance between main and link rings.

VI. INTRODUCING REAL AND IMAGINARY FLUX IN THE RING CHAIN

The dispersion relation (S72) can be expressed in the following way,

$$\omega(k) = \omega_0 + 2t\cos(k-\phi)\cosh h - 2it\sin(k-\phi)\sinh h, \tag{S75}$$

which allows us to inspect the effect of both real and imaginary flux in the system.

Real flux parameterized by ϕ is introduced by displacing the link ring a certain distance y orthogonally with respect to the line between the center of the main rings, see Fig. S5. In essence, this modifies the optical path length of the upper and lower arms of the link ring, and thus induces a phase mismatch between both geometrical paths that approximately amounts to $2\beta y$, with β being the propagation constant of the link ring mode. This translates into a real phase ϕ in the coupling. From (S75), one can see that the real flux shifts both the real and imaginary parts of the spectrum along the k axis, as we showcase in Fig. S7(a) and (b), respectively. Therefore, this shift allows to properly establish a relation between the flux intensity and the displacement y of the link rings. Namely, for the fluxes shown in the figure, we employ a displacement of $y = 0.169\,\mu\text{m}$ (π phase) and $y = 0.169/3\,\mu\text{m}$ ($\pi/3$ phase).

To introduce the imaginary flux, we use the split gain/loss structure explained in Section 3 in the main text. To quantify the asymmetry with respect to this gain and loss, we set $\phi = 0$ and check that the imaginary part of (S75) has a maximum (minimum) at $k = -\pi/2$ ($k = \pi/2$). Comparing $\mathrm{Im}(\omega)$ at these two points yields $\Delta\omega \equiv \mathrm{Im}(\omega(-\pi/2)) - \mathrm{Im}(\omega(\pi/2)) = 4t \sinh h$. Therefore, if we already computed the coupling t according to Supplementary Section V, we can extract the parameter h from

$$h = \mathrm{asinh}\left(\frac{\Delta\omega}{4t}\right). \tag{S76}$$

Alternatively, by knowing that the reverse circulation experiences opposite fluxes, we can apply $h \to -h$ and $\phi \to -\phi$ to (S75). After doing this, we see that the position of the maximum and minimum for the imaginary part are reversed, so we can obtain the same result by comparing $\mathrm{Im}(\omega)$ of both circulations at $k = \pi/2$. From (S76), it is straightforward to compute the asymmetry ratio of couplings $\alpha \equiv t_-/t_+ = e^{-2h}$ for the same circulation, which gives a notion of how close the system is to true unidirectionality.

VII. LOSSY RING RESONATORS

Alternatively to the split gain and loss distribution in the link rings, one may also obtain an asymmetric coupling by employing only lossy rings. The main idea is similar: the upper half of the link ring allows light to propagate and couple to the other rings, while the lower half suppresses this propagation with losses. In this situation one must note that, instead of (S68), now that relation becomes

$$\begin{pmatrix} E_f \\ E_e \end{pmatrix} = \begin{pmatrix} e^{i(\beta\pi R_2+\phi)} & 0 \\ 0 & e^{i(-\beta\pi R_2+\phi)}e^{h} \end{pmatrix} \begin{pmatrix} E_c \\ E_d \end{pmatrix} = M_2 \begin{pmatrix} E_c \\ E_d \end{pmatrix}, \tag{S77}$$

to properly describe the lack of gain in the upper path. Repeating the calculation of the dispersion relation described in Supplementary Section IV now yields

$$\omega(k) = (\omega_0 - iA) + \frac{t}{\cosh(h/2)}\left[e^{h/2}e^{i(\phi-k)} + e^{-h/2}e^{-i(\phi-k)}\right], \tag{S78}$$

where $A = 2t\tanh(h/2)$. It is implicit in (S78) that, aside from the appearance of a lower coupling $t/\cosh(h/2)$, compared to the case with balanced gains and losses, the effective system also displays losses in the rings with a strength that is proportional to the coupling. As we see in Fig. S8, this distorts the bands along the complex plane and the inner bands no longer connect at $k = 0$. This can be explained by the fact that not all main rings are connected with the same number of link rings, so there are different sets of losses in the effective system. However, in the same figure we also observe that this is not enough to break the three-fold splitting of the bands, which is still maintained. For small gain/loss imbalance in the original implementation, only a small distortion of the bands is expected to occur.

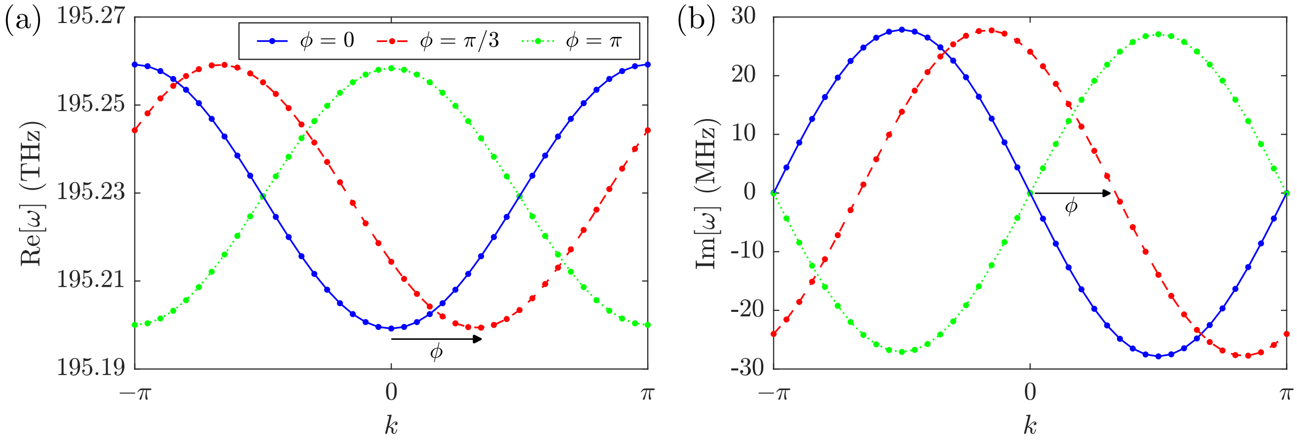

Figure S7. Effect of the real flux on the real part (a) and imaginary part (b) of the eigenfrequencies of the basic MR-LR-MR block (see Fig. S5) for the counterclockwise circulation. The reverse circulation will experience the effect of an equal but opposite flux.

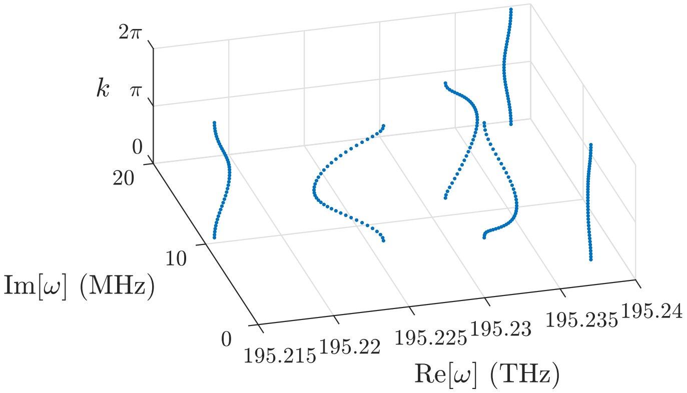

Figure S8. Bulk eigenfrequencies for a periodic chain of the $\sqrt[3]{\text{SSH}}$ model using only losses in the link rings. Compared to the results in Fig. 3(a) of the main text, the bands are distorted and shifted along the complex plane, but the main qualitative features of the three-branch spectrum are still present.

[1] A. M. Marques and R. G. Dias, Generalized lieb's theorem for noninteracting non-hermitian n-partite tight-binding lattices, Phys. Rev. B **106**, 205146 (2022).

[2] P. Delplace, D. Ullmo, and G. Montambaux, Zak phase and the existence of edge states in graphene, Phys. Rev. B **84**, 195452 (2011).

[3] A. M. Marques and R. G. Dias, Analytical solution of open crystalline linear 1d tight-binding models, Journal of Physics A: Mathematical and Theoretical **53**, 075303 (2020).

[4] A. Mostafazadeh, Pseudo-hermiticity versus pt symmetry: The necessary condition for the reality of the spectrum of a non-hermitian hamiltonian, Journal of Mathematical Physics **43**, 205 (2002).

[5] D. C. Brody, Biorthogonal quantum mechanics, Journal of Physics A: Mathematical and Theoretical **47**, 035305 (2013).

[6] F. K. Kunst, E. Edvardsson, J. C. Budich, and E. J. Bergholtz, Biorthogonal bulk-boundary correspondence in non-hermitian systems, Phys. Rev. Lett. **121**, 026808 (2018).

[7] E. J. Bergholtz, J. C. Budich, and F. K. Kunst, Exceptional topology of non-hermitian systems, Rev. Mod. Phys. **93**, 015005 (2021).

[8] K. Kawabata, K. Shiozaki, M. Ueda, and M. Sato, Symmetry and topology in non-hermitian physics, Phys. Rev. X **9**, 041015 (2019).

[9] F. Schindler, K. Gu, B. Lian, and K. Kawabata, Hermitian bulk – non-hermitian boundary correspondence (2023), arXiv:2304.03742 [cond-mat.mes-hall].

[10] A. M. Marques and R. G. Dias, One-dimensional topological insulators with noncentered inversion symmetry axis, Phys. Rev. B **100**, 041104(R) (2019).

[11] L. Madail, S. Flannigan, A. M. Marques, A. J. Daley, and R. G. Dias, Enhanced localization and protection of topological edge states due to geometric frustration, Phys. Rev. B **100**, 125123 (2019).

[12] N. Hatano and D. R. Nelson, Localization transitions in non-hermitian quantum mechanics, Phys. Rev. Lett. **77**, 570 (1996).

[13] W. Deng, T. Chen, and X. Zhang, nth power root topological phases in hermitian and non-hermitian systems, Phys. Rev. Research **4**, 033109 (2022).

[14] D.-S. Ma, Y. Xu, C. S. Chiu, N. Regnault, A. A. Houck, Z. Song, and B. A. Bernevig, Spin-orbit-induced topological flat bands in line and split graphs of bipartite lattices, Phys. Rev. Lett. **125**, 266403 (2020).

[15] M. Ezawa, Systematic construction of square-root topological insulators and superconductors, Phys. Rev. Research **2**, 033397 (2020).

[16] T. Mizoguchi, Y. Kuno, and Y. Hatsugai, Square-root higher-order topological insulator on a decorated honeycomb lattice, Phys. Rev. A **102**, 033527 (2020).

[17] T. Mizoguchi, T. Yoshida, and Y. Hatsugai, Square-root topological semimetals, Phys. Rev. B **103**, 045136 (2021).

[18] Z.-G. Geng, Y.-G. Peng, H. Lv, Z. Xiong, Z. Chen, and X.-F. Zhu, Square-root-like higher-order topological states in three-dimensional sonic crystals, Journal of Physics: Condensed Matter **34**, 104001 (2021).

[19] S. Palmer, Y. Ignatov, R. V. Craster, and M. Makwana, Asymptotically exact photonic approximations of chiral symmetric topological tight-binding models, New Journal of Physics **24**, 053020 (2022).

[20] K. Roychowdhury, J. Attig, S. Trebst, and M. J. Lawler, Supersymmetry on the lattice: Geometry, topology, and spin liquids (2022), arXiv:2207.09475.

[21] E. Edvardsson, F. K. Kunst, T. Yoshida, and E. J. Bergholtz, Phase transitions and generalized biorthogonal polarization in non-hermitian systems, Phys. Rev. Res. **2**, 043046 (2020).

[22] Z. Lin, S. Ke, X. Zhu, and X. Li, Square-root non-bloch topological insulators in non-hermitian ring resonators, Opt. Express **29**, 8462 (2021).
[23] M. Hafezi, S. Mittal, J. Fan, A. Migdall, and J. M. Taylor, Imaging topological edge states in silicon photonics, Nature Photonics **7**, 1001 (2013).
