# Supplementary figures and images for: Topological n-root Su–Schrieffer–Heeger model in a non-Hermitian photonic ring system
Source: Nanophotonics. 2024 Jan 3;13(1):51–61. doi: 10.1515/nanoph-2023-0590 (PMC11501127; doi:10.1515/nanoph-2023-0590)

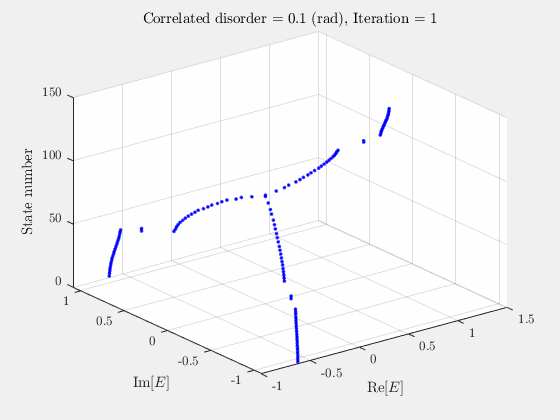

Supplement: Supplementary file 2 — Supplementary Material Details [file j_nanoph-2023-0590_suppl_002.gif]

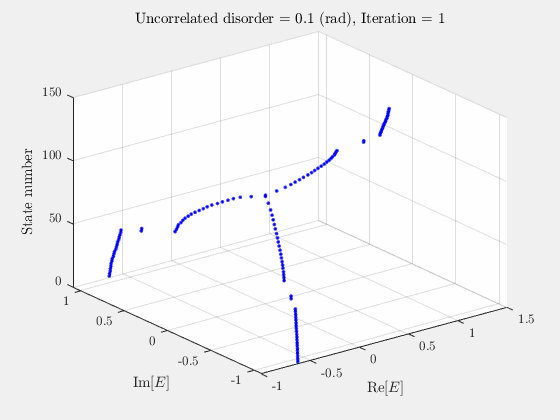

Supplement: Supplementary file 3 — Supplementary Material Details [file j_nanoph-2023-0590_suppl_003.gif]
